# What drives public attitudes towards moral bioenhancement and why it matters: an exploratory study

**DOI:** 10.1186/s12910-021-00732-1

**Published:** 2021-12-09

**Authors:** Marina Budić, Marko Galjak, Vojin Rakić

**Affiliations:** 1 Centre for Philosophy, Institute of Social Sciences, Kraljice Natalije 45, 11 000 Belgrade, Serbia; 2 Centre for Demographic Research, Institute of Social Sciences, Belgrade, Serbia; 3 Centre for Philosophy, Institute of Social Sciences, Center for the Study of Bioethics, Belgrade, Serbia

**Keywords:** Ethics, Bioethics, Enhancement, Public attitudes, Deontology, Utilitarianism

## Abstract

The paper represents an empirical study of public attitudes towards moral bioenhancement. Moral bioenhancement implies the improvement of moral dispositions, i.e. an increase in the moral value of the actions or character of a moral agent. The views of bioethicists and scientists on this topic are present in the ongoing debate, but not the view of the public in general. In order to bridge the gap between the philosophical debate and the view of the public, we have examined attitudes towards moral bioenhancement. The participants were people from Serbia older than 15, who voluntarily completed an online questionnaire. The questionnaire consisted of a brief introduction to moral bioenhancement, seven general questions, 25 statements about participants’ attitudes towards moral bioenhancement, and five examples of moral dilemmas. The questionnaire also included questions which were used to reveal their preference of either deontology, or utilitarianism. Participants were asked to what degree they agree or disagree with the statements. The results showed that the means used to achieve moral enhancement, the level of education, and preference for deontology or utilitarianism do have an impact on public attitudes. Using exploratory factor analysis, we isolated four factors that appear to drive the respondents' attitudes toward moral bioenhancement, we named: general—closeness, fear of change, security, and voluntariness. Each factor in relationship to other variables offers new insights that can inform policies and give us a deeper understanding of the public attitudes. We argue that looking into different facets of attitudes towards moral bioenhancement improves the debate, and expands it.

**Supplementary Information:**

The online version contains supplementary material available at 10.1186/s12910-021-00732-1.

## Background

This paper represents a part of an empirical study^1^ of public attitudes towards moral bioenhancement. Moral bioenhancement (MBE) refers to using medical, pharmacological, or biotechnological means to improve moral dispositions and motives. Given the rapid evolution of society and technology, the discourse on ethics is becoming increasingly relevant. The issue of MBE is important, since moral behavior and humanity are, along with rationality, precious and essential values that define us as human beings. While there is a considerable discussion about the moral propriety of MBE, the views of the public have largely been absent from the discussion. We explore people’s attitudes towards MBE, to either confirm or raise doubts regarding some findings of a previous study [1], but also to start a discussion about the major factors that drive attitudes toward MBE that have not been isolated before.

We were interested in the different facets of why people (dis)approved of MBE. Given that morality at the societal level is contextual, we highlight the importance of querying and understanding a part of the public in Serbia on this issue, and seeing whether the results cohere with the previous US study [1].^2^

Our study represents a part of the population of one country and the sample is not fully representative of the entire population of Serbia. The respondents consisted of people who voluntarily filled out an online questionnaire about MBE. Nevertheless, our study gives certain indications and directions in which manner to conduct future studies on this topic.

Our approach is inspired partly by discussions of philosophers and scholars on MBE, and partly by *experimental philosophy* [2, 3]. This approach can help us understand how people think about the ethical issues of MBE, which could be useful for developing policies. Doris et al. [4] pointed out that results from experimental philosophy reveal people’s intuitions and biases on a subject, which can help philosophers avoid their own biases.

### Moral bioenhancement

*Moral enhancement* (ME) of a moral agent represents an increase in the moral value of their actions or character, i.e., the improvement of their moral dispositions, rather than physical and/or cognitive capacities. MBE implies achieving this by using medical, pharmacological, or biotechnological means. MBE is an intervention for people to biomedically mitigate their counter-moral emotions [5]. While ME includes any means of moral improvement, MBE only involves biomedical interventions [6].

Although the scenarios proposed in philosophical thought experiments still belong in the realm of science fiction, there are examples of drugs and methods that could be considered as a form of MBE.

Drugs such as Propranolol can reduce implicit racial bias, and produce less utilitarian judgment [7], Ritalin, Adderall, and other drugs improve impulse control in children with attention deficit disorder, and indeed reduce violence and antisocial behavior [7]. Savulescu et al. [7] point out that oxytocin mediates maternal care, pair bonding, and other prosocial attitudes like trust [8], trustworthiness [9], empathy [10], and generosity [11]. Selective serotonin reuptake inhibitors (SSRIs) make subjects more fair-minded and willing to cooperate [7]. Enhanced serotonin function increases aversion toward harming self and others, whereas enhanced dopamine levels decrease feelings of harming oneself over others [12]. Other possible techniques for influencing choices include Transcranial Magnetic Stimulation, Deep-Brain Stimulation, Transcranial Direct Current Stimulation [13], and Optogenetics, which can directly modify behaviors, perhaps even addictive behavior [14].

The motivation for the idea of MBE is rooted in the following considerations. Namely, the proponents of MBE [7, 15] argue that the traditional means of moral education and socialization are not satisfactory for humanity to deal with the ethical problems in modern society, so there is a need for bioenhancers. Authors [7, 16, 17] have proposed that we should explore the possibility of biologically enhancing our morality, rather than continuing to rely upon ineffective methods. Besides, Persson and Savulescu [15, 18] emphasize that it is much easier to harm than to benefit, and also that cognitive enhancement (CE) and technological progress enable humans to use means of mass destruction, which is very dangerous without moral progress and ME. Further, Frank [19] argued that *technologies to improve people's moral capacities are realizable*, while Conan [20] recently defended the efficacy and practical and ethical feasibility of MBE interventions.

However, there are concerns about the ethics of MBE [21]. Azevedo [22] argues that we still don’t have enough reasons to adhere to Persson’s and Savulescu’s pessimism of our inaptitude to face the challenges of our near future without the aid of radical means of ME. Balisteri [23] argues that hopes and expectations in MBE should be strongly reconsidered and debunked because morality is a kind of experience that cannot be construed just through technologies. He argues that the fact that we could remove a lot of sentiments or emotions that prevent correct practical reasoning is just the preliminary condition for the agent to act morally and, above all, to recognize the moral unacceptability of certain behaviors [23]. Sparrow [24] maintains that common drugs such as alcohol, MDMA, and cannabis are also capable of making people *more moral*. He believes that the claim—that changing people’s behavior and emotions is sufficient to constitute MBE—presupposes a consequentialist view of what makes people *more moral*, and that virtue ethics and Kantian view of the nature of morality require something more than mere behavior alterations [24].

### Previous studies

There are only a couple of studies on the moral propriety of using bioenhancers, and all the studies have been conducted in the US. A study about people’s attitudes toward CE [25] suggests that the public is sensitive to and capable of understanding the main concerns identified by neuroethicists, and shows support for both transhumanist and bioconservative views. Another piece of research concerning people’s attitudes toward MBE in the US [1] shows that the public disapproves of biomedical interventions for ME, yet is open to non-biomedical means to realize ME, i.e. means of achieving ME matter morally.

Participants (N = 293) from the US were randomly assigned to read one of several contrastive vignettes in which a 13-year-old child is described as bullying another student in school and then is offered an empathy-enhancing program [1]. The program either involve taking a pill or playing a video game. Also, participants were asked to imagine their own child either bullying another student, or being bullied by another student. The authors used the contrastive vignette technique (the two contrasts were pharmacological or non-pharmacological means, and the closeness to the subject: other’s child or own child), and employed a novel mixed-methods design in which content analysis of free-response answers were quantitized and assessed in a contrastive fashion [1].

Results showed that the respondents were significantly less supportive of a mandatory pharmacological than a mandatory non-pharmacological anti-bullying program. People were less supportive of empathy enhancement within the context of prevention of future immoral behaviour as compared to support for empathy enhancement in cases where immoral behaviour has already manifested itself [1].

Further results indicated that respondents supported a mandatory preventive empathy-enhancing program for all children that involved playing a video game more than one that involved taking a pill. Thus, respondents were significantly less supportive of requiring all children to participate in a mandatory pharmacological empathy-enhancing program compared to their support for required participation in a non-pharmacological program. Taken together these results indicate that people were consistently more troubled by pharmacological than non-pharmacological moral enhancement interventions [1].

Riis et al. [26] found that young, healthy individuals were much more reluctant to enhance traits believed to be more fundamental to self-identity than the traits considered less fundamental to self-identity.

## Methods

### Aim

Our research aimed to replicate findings of the previous study done in the US [1], but also to identify the factors that underlay the responses which were crucial in assessing the propriety of MBE. Also, our goal was to test if the level of education and familiarity with the concept impacted people’s attitudes about MBE. Therefore, we aimed to test a range of issues that were central to the MBE debate. To that end, we formed four testable hypotheses, stated below.

**H1 **The degree to which members of the public support an ME program depends on whether the means employed are pharmacological or non-pharmacological.

**H2** The level of support for MBE differs, depending on people’s familiarity with the concept of *MBE*.

**H3** People’s attitudes towards MBE differ depending on people’s levels of education.

**H4** The degree to which someone is prone to utilitarian reasoning is correlated with that person’s attitudes toward MBE.^3^

In addition to the stated hypotheses, we set out to explore the responses for any simple, interpretable, underlying factor structure that might emerge from the questions directly referring to MBE.

### Procedure

The survey was posted online via *online forms*, and the participants were people who voluntarily completed the questionnaire. The age limit for participants was predefined at 15 years (first year of high school in Serbia), as the concepts in the questionnaire were too advanced for elementary school students. The first part of the questionnaire consisted of a brief introduction to MBE and seven general questions (sociodemographic, and question on familiarity with the term MBE). Subsequently, we used 25 statements (based on the literature review) in order to examine participants’ attitudes towards MBE. Participants were asked to rank to what degree they agreed or disagreed with the statements, ranging from 1 (not at all) to 7 (completely).

#### Example of a question

I think moral bioenhancement should be mandatory for criminals. (e.g., a pill that increases empathy and a sense of justice).

The second part of the questionnaire consisted of five examples of moral dilemmas.^4^ Then, participants were presented with a series of questions and used the same 7-point Likert scales to respond.

#### Example of a moral dilemma

Imagine that your child is a victim of peer violence. Every day, your child suffers physical abuse, while on social media, violence continues through belittling, insulting, and telling lies about your child. The school has a program that has been proven to be effective in reducing peer violence through carefully carried out studies. The program involves the following: over the course of 4 weeks, each day the bully takes a pill that increases empathy for others. The pill is based on the natural hormone oxytocin and improves the bully’s ability to understand what other people are feeling. Several studies have shown that the program significantly reduces peer violence without any negative side effects. The effects of the pill are visible for several months after the program is complete.

#### Example of a question related to the dilemma

To what degree do you think that it would be a good idea for the bully to participate in a program like the one described above?

Finally, five questions referred to the participants’ preferences for either deontology or utilitarianism. Kahane et al. [27] investigated dimensions of utilitarian thinking and developed a new scale that is both philosophically rigorous and empirically driven, and attempts to address the concerns about sacrificial dilemmas and the existing scales. We have used the four items from their research we consider to be the most relevant and representative, in order to contrast utilitarian and deontological judgments, and one classical deontology/utilitarianism statement—*The end justifies the means*. Thus we developed a scale to investigate whether the preference for deontological or utilitarian reasoning influenced people’s attitudes towards MBE. The survey included additional questions that were not part of this study. For the complete list of questions see the Additional file 1: Text S1.

### Statistical analysis

Data were analyzed using R [28] with psych [29], rstatix [30], and epitools [31] packages. The scripts necessary for reproducing the analysis, together with the data, are included in the supplementary material. The general attitude towards MBE was assessed by creating a composite score based on the questions where respondents needed to declare their support for a proposition concerning MBE. The responses to questions were not normally distributed (as evaluated by the Kolmogorov–Smirnov test and Q-Q plots of the data), which was why we opted for the nonparametric statistical methods in testing the hypotheses. We performed exploratory factor analysis (EFA) to narrow down the dimensions and discover patterns underlying the variables from the questionnaire. The EFA was performed on a subset of questions that were strictly related to MBE. Good example of the criterion for the selection of questions were the questions provided the scenario where a child was bullied in school. The respondents were asked if the bully should be offered a pill to improve their behaviour, and for another different question we provided the same scenario but with video games as means of improving their behaviour. The former was included in the EFA, but not the latter, as it represented ME rather than MBE. The comprehensive list of questions included in the EFA is provided in the Additional file 1: Table S1. The details about each aspect of the statistical analysis are given below, and the code and the data needed to replicate the analysis are in the supplementary materials. The data itself are also deposited in a public repository [32].

### Exploratory factor analysis

We performed exploratory factor analysis (EFA) using the MinRes method, to find a solution with minimal residuals [33] on the covariance matrix encompassing all the questions directly related to MB (30 of them), including both the questions based on concrete examples and direct, declarative questions. The Kaiser–Meyer–Olkin (KMO) factor adequacy measure showed that the proportion of possible common variance among variables was very high (the overall MSA = 0.93) [34], and the internal consistency of the question set was confirmed using Cronbach's alpha (α = 0.86) [35]. The number of common factors to retain was determined considering: scree plot (Additional file 1: Figure S1), eigenvalues, parallel analysis, and ultimately, by interpretability of the solution [36]. The fit of the solutions was evaluated using the Tucker Lewis Index of factoring reliability (TLI) [37], the comparative fit index (CFI) [38], and the root-mean-square error of approximation (RMSEA).

The strength of the independent association of extracted factors and selected characteristics of the respondents were quantified using odds ratios with Yate's continuity correction. For each factor, we calculated the odds ratios for being above the median factor score.

### Composite scores

The questionnaire with all questions (and answers to scenarios) provided us with 59 variables, and one topic was featured in multiple questions. To make the hypotheses testing more robust, we created composite scores that combine several variables covering the same topic. This was preferred to picking a question to test a hypothesis, as a more robust approach. We initially created 4 variables that grouped similar questions (covering the same topic) around—Pharmacological means (MBE) score, Non-pharmacological means (ME) score, Total MBE Support score (with all the questions where the support for MBE was examined) and Utilitarianism score (with the questions which examined deontology/utilitarianism preference). We measured internal consistency and reliability with Cronbach’s alpha and Guttman's Lambda 6. We also considered signal to noise ratio, in estimating usefulness of the composite score. One of the composites, grouping preferences for deontology-utilitarianism was not satisfying (Table 1) so we discarded it. As four of the questions were directly taken from Kahane et al. [27] study, picking any one of those would cover only one of the factors that they have identified (Impartial Beneficence or Instrumental Harm). Instead, we selected responses to the general statement (The end justifies the means) to represent the utilitarian scale.

**Table 1:** Internal consistency and reliability of the composite variables

Composite variable	Number of questions	Cronbach’s α	Guttman's 6 λ	Signal/Noise
Pharmacological means score	7	0.91	0.93	10
Non-pharmacological means score	7	0.93	0.94	13
Total MBE Support score	29	0.84	0.93	5.3
Utilitarianism Score	5	0.36	0.36	0.48

The detailed list of items included in each composite variable is provided in the Additional file 1: Table S2.

### Hypothesis testing

The hypotheses were tested using single variables and composite scores.

For the hypotheses where the null hypothesis was that there is no difference in the medians for two variables, the Wilcoxon two-sample paired signed-rank test was used. In this case, the result was reported using p-values together with effect size *r* and its 95% confidence interval and the W test statistic. This test was used for hypothesis H1.

For the hypothesis where respondents were grouped by some variable to test whether the groups had the same median value, the Wilcoxon–Mann–Whitney test was used. In this case, the result was reported by using p-values together with effect size *r* and its 95% confidence interval and the U test statistic. This test was used for hypothesis H2.

For testing whether the attitudes towards MBE differed among different groups, we used the Kruskal–Wallis rank-sum test. Post-hoc pairwise comparisons were performed by using Dunn’s test. The results were reported with chi-squared (${\chi }^{2}$) statistics and p-values, while the results of Dunn’s test were reported by using the Holm–Bonferroni adjusted p-values. This test was used for hypothesis H3.

For testing hypotheses on the correlation between two variables, we used the Kendall rank correlation coefficient. The result of the correlation test was reported by using p-values together with the correlation coefficient *τ*. This kind of test was used for hypothesis H4.

## Results

### Sample

The sample included 337 participants from Serbia (30% male, 70% female), and their average age was 38.05 (SD = 12.9, min = 15, max = 84). The sample skewed towards more educated, urban population, as more than a third of participants had advanced degrees and the vast majority lived in urban areas. See Table 2 for the detailed descriptions.

**Table 2:** Sociodemographic characteristics of the participants (N = 337)

	N	%
Sex		
Male	102	30.27
Female	235	69.73
Age		
< 25	58	17.21
25–29	78	23.15
30–34	70	20.77
35 +	131	39.87
Education		
No degree	110	32.64
Bachelor's degree	101	29.97
Advanced degree	126	37.39
Dwelling		
Rural	43	12.76
Urban	294	87.24

### Exploratory factor analysis

**Fig. 1:**
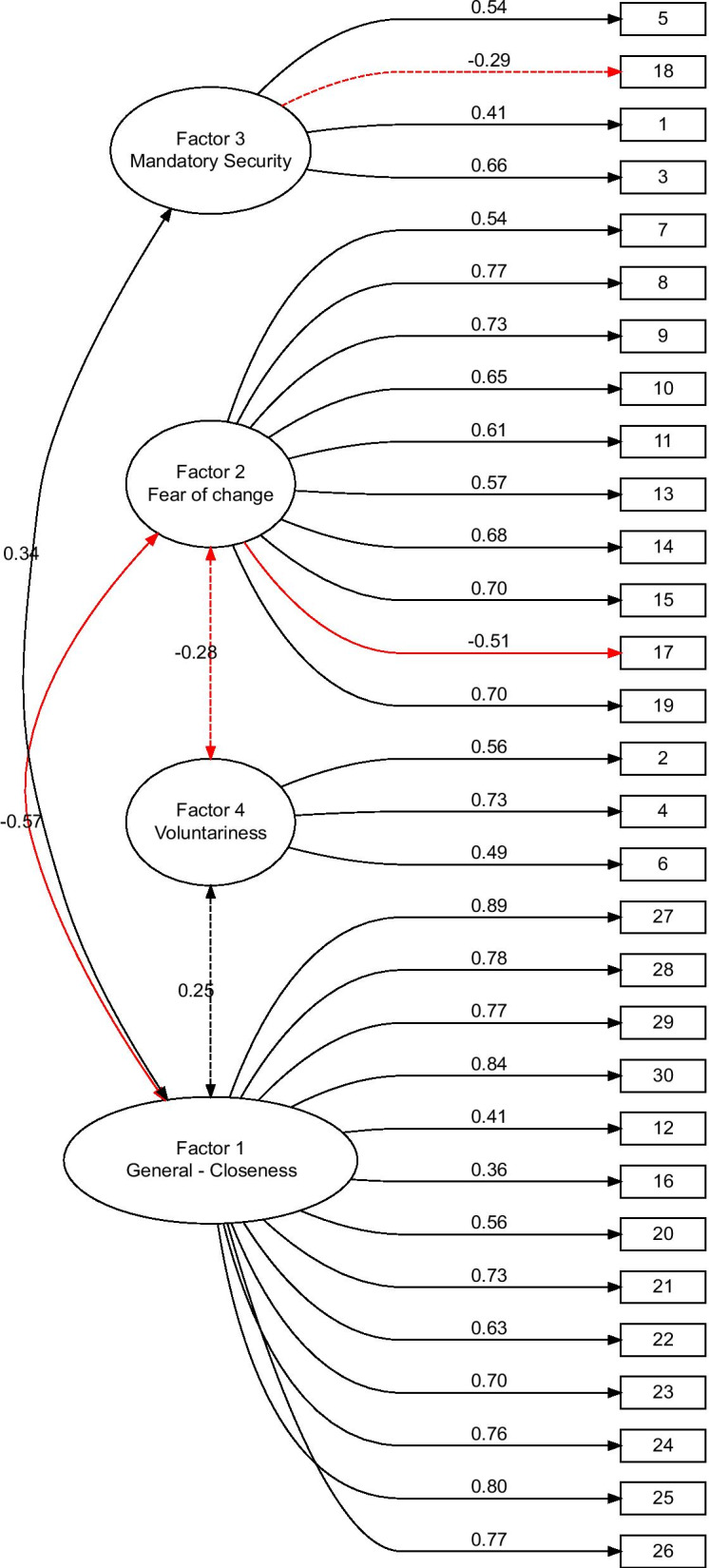
Four-factor model

Based on the multiple criteria, including the scree plot method (Additional file 1: Figure S1), we determined that the optimal number of common factors to be extracted was four. The solution with four factors was rotated using an oblique method—*oblimin* [39]. The oblique rotation was preferred over the orthogonal, since we expected common factors to be mutually correlated. The solution has one factor that was negatively correlated with the others (Fig. 1). The fit of the solution was evaluated using the Tucker Lewis Index of factoring reliability (TLI = 0.951), comparative fit index (CFI = 0.964), and the root-mean-square error of approximation (RMSEA index = 0.085 95%CI [0.079, 0.09]) (Table 3). Although the solution broke the convention that RMSEA should be below 0.08, we opted for the four-factor solution because the five-factor solution (which satisfies this convention) was more complex to interpret and added just 2% to the explained variation (Table 4) [40].

**Table 3:** Model summary (all items)

Number Of Factors	Cumulative variance (%)	${\chi }^{2}$	TLI	CFI	RMSEA
1	42	2,594.22 (405)	0.892	0.899	0.127 [0.122, 0.132]
2	49	1,794.04 (376)	0.924	0.934	0.106 [0.101, 0.111]
3	54	1,417.14 (348)	0.938	0.951	0.095 [0.09, 0.101]
**4**	**58**	**1,095.41 (321)**	**0.951**	**0.964**	**0.085 [0.079, 0.09]**
5	60	832.41 (295)	0.963	0.975	0.073 [0.068,0.08]
6	62	695.62 (270)	0.968	0.980	0.068 [0.062, 0.075]

The selected model is bold

TLI, Tucker–Lewis index; CFI, comparative fit index; RMSEA, root mean square error of approximation

**Table 4:** Extracted factors of the four-factor model by the percentage of variance they explain

Factor	Name	Variance (%)	Cumulative variance (%)	Cumulative factor variance (%)
1	General—closeness	29	29	50
2	Fear of change	16	45	77
3	Mandatory security	8	52	90
4	Voluntariness	6	58	100

### Factor descriptions

Factor 1—*General—Closeness* is best determined by questions that *hit close to home.* The questions with the heaviest loadings were those related to children and the cases where the respondent needed to identify with a scenario. This factor explained 29% of the variation, which was more than any other factor, accounting for a half of the total variation explained by all the extracted factors combined (Table 2). The prefix *general* in the name of the factor was due to the fact that it carried the most weight and determined the general attitude the most. Someone’s support for bioenhancement would be heavily influenced by the context, i.e., by how close to home it hit. This was based on the distinction between self and other.

Factor 2—*Fear of change* was determined by the variables that questioned the means of MBE, as well as the clashes with free will, identity, and tradition. It was the second most important factor, and it explained 16% of the variation. This factor was the easiest to interpret, with the heaviest loadings being on the question which explicitly proposed the following: “*MBE is unethical because the means for its attainment are unethical”.* The pharmacological nature of many proposed scenarios and *unnaturalness* particularly drove this factor. This factor was strongly negatively correlated with the first factor (*r* = − 0.57), meaning that the more someone was scared of the means, the less comfortable they would generally be with potential MBE.

Factor 3—*Mandatory Security* was mostly determined by the questions regarding the mandatory, population-wide interventions, the purpose of which would be to increase security by way of MBE. This factor accounted for 8% of the variation. This factor was mostly driven by the questions revolving around propositions that posited utilizing MBE to prevent crime, bullying and general increase in security. It was moderately correlated with the first factor describing General—Closeness(*r* = 0.34), and weakly negatively correlated with the second factor (*r* = − 0.11).

Factor 4—*Voluntariness* was exclusively determined by the variables that questioned bioenhancement as an allowed option for the people who *chose to take the pill.* This factor explained the least variance (6%) and was moderately correlated with factor 1 (*r* = 0.25), similarly negatively correlated with factor 2 (*r* = − 0.28), and weakly correlated with factor 3 (*r* = 0.12). The three questions, which had big loadings with this factor, were directly covering the *voluntary aspect*, but some questions had the *voluntary aspect* while loading higher on other factors.

In Table 5 we presented the calculated odds ratios for each factor by age, sex, education, and two other variables—one based on the question which reads “Are you familiar with the concept of MBE”, the other describing how utilitarian they were (the grouping was made using the median composite score created using the last four questions from the questionnaire to split the sample into the two groups).

**Table 5:** Odds ratios for extracted factors by selected variables

	Factor 1 (General—closeness)	Factor 2 (Fear of change)	Factor 3 (Mandatory security)	Factor 4 (Voluntariness)
Age				
< 25	1	1	1	1
25–29	0.54 [0.27, 1.07]	0.61 [0.30, 1.21]	0.38** **[0.18,0.77]**	1.41 [0.71, 2.81]
30–34	0.62 [0.30, 1.26]	0.67 [0.33, 1.35]	0.68 [0.33, 1.67]	1.15 [ 0.57, 2.32]
35 +	0.63 [0.33, 1.18]	0.68 [0.36, 1.26]	1.05 [0.56, 1.96]	1.06 [0.57, 1.99]
Male	0.95 [0.60, 1.52]	1.13 [0.71, 1.80]	0.57** **[0.36, 0.92]**	1.19 [0.75, 1.91]
Education				
No degree	1	1	1	1
Bachelor's degree	0.56* **[0.32,0.97]**	0.69 [0.40, 1.19]	0.89 [0.52, 1.54]	1.45 [0.84, 2.51]
Advanced degree	0.44** **[0.26, 0.73]**	1.09 [0.65,1.82]	0.55* **[0.32, 0.92]**	0.89 [0.53, 1.49]
Familiar with the concept of moral bioenhancement	0.90 [0.55, 1.45]	0.59* **[0.36, 0.95]**	0.94 [0.59,1.54]	1.14 [0.70, 1.84]
Utilitarian reasoning	2.41*** **[1.56, 3.75]**	0.63* **[0.41, 0.97]**	1.88** **[1.22, 2.91]**	1.01 [0.66, 1.55]

Odds ratios were calculated for being above the median value of the factor score. 95% CI is in the brackets

Odds ratios with significant *p*-values are bold

*p < 0.05; **p < 0.01; ***p < 0.001

### Hypotheses

**Fig. 2:**
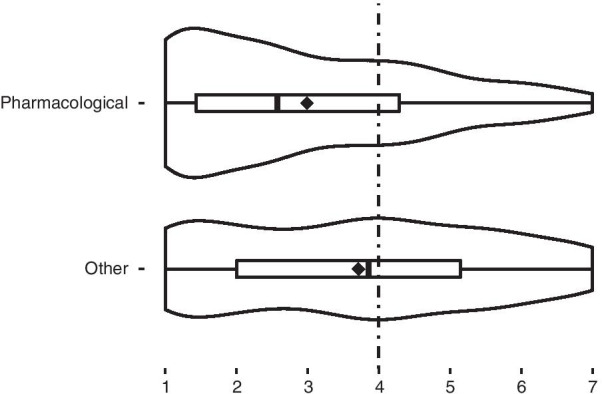
Support for moral enhancement based on the means of achieving it. ♦, mean value for each distribution

**H1** has been confirmed. The answers to the questions where the only difference concerned the means, i.e., whether the means were pharmacological or non-pharmacological, largely varied (r = 0.728 95%CI [0.68, 0.78], W = 34,206, *p* < 0.001), where the respondents were more in favor of non-pharmacological means (Fig. 2).

**Fig. 3:**
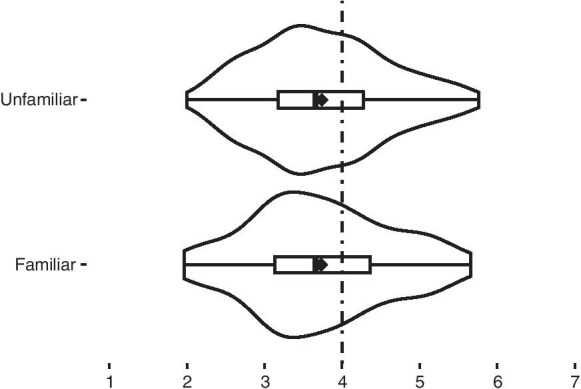
Support for moral bioenhancement among the people who said they were familiar with the concept and those who said they were not. ♦, mean value for each distribution

**H2** has not been confirmed. Respondents who were familiar with the concept of *MBE* were neither more nor less supportive of MBE (*r* = 0.025 95% CI [0.002, 0.14], U = 11,182, *p* = 0.644) (Fig. 3).

**Fig. 4:**
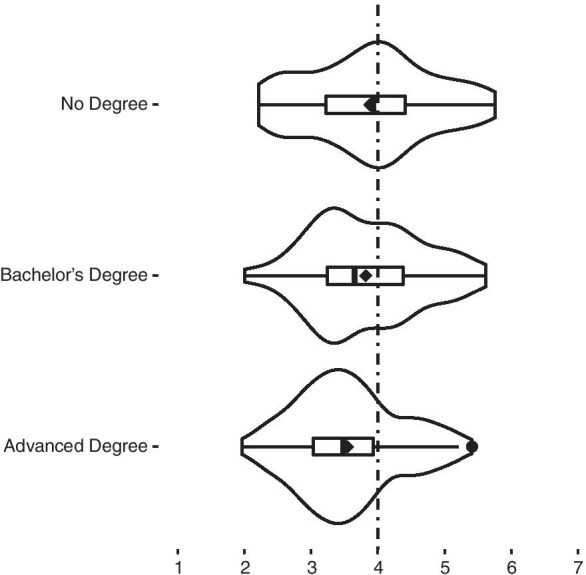
Support for moral bioenhancement based on education. ♦, mean value for each distribution

**H3** Respondents with different levels of education showed different attitudes towards MBE (${\chi }^{2}$(2) = 10.9, *p* < 0.004). People with no degree showed the most support for MBE (Me = 3.93), the ones with a four-year Bachelor’s degrees showed less support for MBE (Me = 3.66), while those with advanced degrees showed the least support for MBE (Me = 3.48) (Fig. 4). Post-hoc Dunn’s test showed that only the group with advanced education stood out significantly compared to those with no degree (adj. *p* < 0.006), but also compared to those with Bachelor’s degree (adj. *p* < 0.03).

**H4** has been confirmed. Respondents who were more utilitarian oriented had more positive attitudes toward MBE (*τ* = 0.198, *p* < 0.001).

## Discussion

### Factors

#### Factor 1—*general—closeness*

This is the factor that explains most of the variance in the responses. It is important for future studies of attitudes towards MBE. These findings must be taken into account—either by posing all questions (and scenarios) in the questionnaire in a general way (third-person) or by composing the items in as way as to isolate the variance coming from this factor.

Specker et al. [1] identified a previous study [41] that found that people had different attitudes concerning the fairness of CE, believing that it was more morally acceptable if they used it than if the other people did. We found that this asymmetry influenced people’s reasoning about MBE interventions in our sample as well.

#### Factor 2—*fear of change*

The isolation of this factor is not surprising, given that similar concerns exist in the bioethical debate on MBE as well. These concerns include identity change, unnaturalness, restricted freedom, and autonomy.

*Identity change.* According to Douglas [5], it could be argued that a person’s MBE would be identity-altering in the sense that it would change some of her most fundamental psychological characteristics.

*Unnaturalness.* This objection implies that MBE is wrong because it is unnatural in the sense it is miraculous or supernatural, rare or unusual, and artificial (for more, see: Douglas [5]).

*Restricted freedom and autonomy.* Although freedom and autonomy are very important issues in bioethical discussions about MBE, they didn’t form a distinct factor. Given that these values are of great importance to human beings and that in philosophy freedom is considered one of the highest values, we investigated whether the public thought these values would be damaged by MBE. Harris [42] argues that MBE could seriously jeopardize and restrict the freedom of the person using them. Authors [43–46] emphasize what they see as threats to human nature, dignity, and freedom as the basis for their concern about enhancement. Simkulet [47] argues that „the taking away of an agent’s free will is one of the greatest harms that can befall a person“. Sparrow [24] claims that the *enhancers* will be wielding power over the *enhanced*. For the discussion on autonomy and MBE, see Protopapadakis [48].

#### Factor 3—*mandatory security*

The isolation of this factor shows that people’s attitudes are at least partially influenced (guided) by the notion of security. The existence of this factor is embedded into the bioethical debate on MBE. For example, involuntary MBE treatment has been justified by appealing to reasons for public security [7, 16]. Walker [49] suggests that MBE could achieve a significant reduction in criminal behavior, such as rape, murder, and torture. Persson and Savulescu [15] indicate that MBE can reduce this risk of death and disaster, as well as the malevolence or viciousness, and protect the security of people. Specker et al. [50] warns that MBE does not equal criminality reduction, and that this false equivalence should not be an argument for MBE.

#### Factor 4—*voluntariness*

The existence of this factor is also part of the current philosophical debate on MBE, and the question of whether ME should be permitted, and if so, whether it should be compulsory or voluntary. Persson and Savulescu [16], for example, do not rule out that MBE could be justifiably imposed without the informed consent of the subjects. They believe that it would be most effective if it is so imposed on children [16]. Crutchfield [51] argues that compulsory MBE should be administered even covertly. Some authors argue in favor of mandatory MBE of psychopaths [52], although some of them are against it [53]. On the other hand, Rakić and Ćirković believe that if MBE were compulsory, it would deprive humans of their freedom, a key component of their human existence [54]. Agar [55] also argues that enhancement should be permissible but not obligatory. Simkulet [47] supports voluntary enhancement, but not compulsory.

### Hypotheses

#### Rage against the pills

**H1** There was a large difference between the answers to the questions where the only difference was concerning the means, i.e., whether the means were pharmacological or non-pharmacological, where respondents were more in favor of non-pharmacological means (Fig. 2). Our result is in accordance with the previous study carried out by Specker, Schermer, and Reiner [1]. Their results indicated that members of the public for the greater part opposed pharmacological MBE [1]. Our studies together show that to the public, the *means* matter morally. The data confirm that the public disapproves of biomedical interventions in ME. Members of the public in the US and also in Serbia are generally disinclined to pharmacological MBE, while people in the US are more open to non-biomedical means of attaining ME than people in Serbia. One interesting finding is that there is no difference in demographic characteristics (such as gender and age) when it comes to the second factor, *Fear of change*, that, among other things, describes the means of achieving the enhancement. This implies that people generally disapprove of MBE, regardless of their gender and age. Authors have different opinions on this issue. Spence [56] argues that taking medicine is not intrinsically moral or immoral, but rather a human subject can use medication as a means to assist them towards a moral end: reducing future harm [56]. Simkulet [47], however, argues that these interventions fail to constitute genuine ME because, although they may result in more desirable outcomes—more altruism, more law-following, and/or less self-destructive behavior, they ignore a person’s intentions, while what often makes an action right or wrong is the intent behind it. Harris [42] argues that the only reliable methods of ME, either now or for the foreseeable future, are socialization, education, and parental supervision, or those high-tech methods that are general in their application. By this, Harris refers to forms of CE that improve cognitive abilities (e.g., improvements of memory, concentration, attention, wakefulness) and do not target specifically *ethical* capacities [42]. Balisteri [23] considers empathy and concludes that *appropriate* empathy cannot be obtained just through new enhancing biotechnologies, because it seems to be strictly related to our capacity to recognize our limits and prejudices.

#### Ignorance is (not) bliss?

**H2** Respondents who were familiar with the concept of MBE were neither more nor less supportive of MBE (Fig. 3). Although H2 has not been confirmed, when we view these results in the context of different factors, we discover there was an interesting dynamic with factor 2—*Fear of change* (Table 5). The familiarity with the concept of MBE was reflected only in the second factor and influenced the fear of MBE. In other words, if someone was familiar with the concept of MBE, they were less likely to be afraid (especially of the means), but this *inoculation effect* was not so strong as to sway them completely one way or the other. This finding shows the importance of factor analysis. These kinds of differences are not visible when looking at global attitudes towards MBE, nor when looking into the specific question, but are only noticeable when broken down into factors.

We made this hypothesis based on literature. *The mere-exposure effect* is a psychological phenomenon by which people tend to prefer something just because they are familiar with it. The examples of this phenomenon include words, Chinese characters, paintings, pictures of faces, geometric figures, and sounds [57–59]. Robert Zajonc has developed the effect [59, 60]. He hypothesized that mere repeated exposure of an individual to a stimulus object enhanced his/her attitude toward it, and the *mere* exposure was a condition making the stimulus accessible to the individual’s perception [60]. Accordingly, more positive attitudes towards MBE were expected to be associated with increased familiarity with the concept. If this is indeed a consideration, then scientists and educators should focus on the education of people about this topic, especially of the younger people.

#### No pills for the elites

**H3** Respondents with different levels of education showed different attitudes towards MBE. People with no degree showed the most support of MBE, the ones with a four-year Bachelor’s degree showed less support of MBE, while those with advanced degrees showed the least support of MBE (Fig. 4). Only the group with advanced education stood out significantly, compared to those with no degree, but also compared to those with Bachelor’s degree.

We expected people with higher education to show more critical attitudes towards MBE, i.e., less support for these interventions. At the same time, people with higher education might be more open to using different means and new technologies. Our results showed that people with an advanced degree indeed manifested more careful and circumspect attitudes towards MBE than people with no degree (Table 5). This can be associated with critical thinking, which is encouraged in the institutions of higher education. This difference is the most conspicuous when it comes to the first factor. Surprisingly, there were no statistically significant differences with the second factor, meaning that fear of the means was present to the same degree no matter how educated the respondents were. When it comes to the third factor, there was a statistically significant difference—those with advanced degrees differed from those with no degree, as the most educated were less likely to support mandated measures to increase security than those the least educated.

The level of education has an impact on people’s attitudes towards different issues. Knoke and Isaac [61] in their study showed that the quality of education affected sociopolitical attitudes, since in five of six sociopolitical attitudes (education, marijuana, abortion, busing for social integration, sex roles, Vietnam, and vote), the quality of higher education was positively correlated with the liberal response. A secondary analysis of the data from the British Social Attitudes showed that higher education often meant: more political engagement, greater concern about the environment, less traditional and more tolerant attitudes to gender equality and immigrants [62]. Previous research suggested that higher education might reduce prejudice and promote egalitarianism and tolerance [63–66]. However, Chatard and Selimbegovic explain that this varies greatly across disciplines, so that some fields of study, such as sociology, humanities and the social sciences promote egalitarianism more than those of business, administration or economy, along with nature of the culture (collectivist and individualist), and socialization [67].

#### The greatest amount of pills for the greatest number of people

**Fig. 5:**
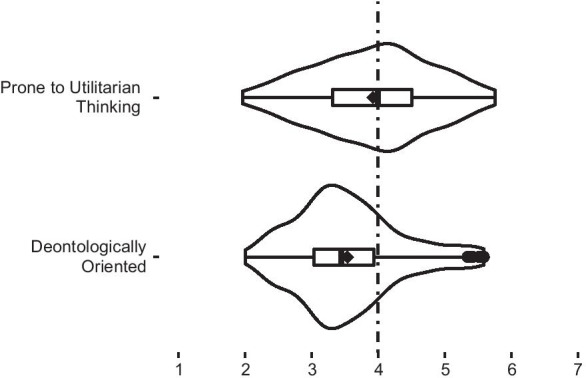
Support for moral bioenhancement based on preference for either deontology or utilitarianism. ♦, mean value for each distribution

**H4** Respondents who were more utilitarian oriented had more positive attitudes toward MBE (Fig. 5). This hypothesis has been confirmed, and there were differences between deontologically oriented participants and utilitarian oriented participants in the three factors. Participants prone to utilitarian thinking in our sample had more positive attitudes towards MBE. However, with the factor 2 the difference between deontologically oriented participants and utilitarian oriented participants was inverted (since this factor was negatively correlated with the general approval of MBE). Not only were the participants prone to utilitarian reasoning more supportive of MBE, but they were less afraid of the means and more likely to support population-mandated MBE.

Based on these theoretical foundations, we expected respondents prone to utilitarian reasoning to show more positive attitudes toward MBE. Deontology and utilitarianism are two of the most prominent approaches to normative ethical theories that deal with the criteria of what is morally right and wrong. Deontology emphasizes the notions of obligations, duties, right, or wrong. Teleological theories (to which utilitarianism belongs, as the paradigmatic case of consequentialism) emphasize the notions of good, desirable, and consequences.^5^

Since deontologists focus more on reasons and motives behind acts, not only on consequences, the manner in which moral enhancement is achieved will be more important than the moral behavior itself. Utilitarian point of view, on the other hand, implies that the consequences and overall good is what are more important. Sparrow [24] argues that people with propensity for consequentialism may evaluate positively and approve any intervention, including taking pills, that makes people more likely to behave in a good way. This will likely not be the case with virtue ethicists and deontologists.

## Limitations

This is an exploratory study. The sample is not fully representative of the population of Serbia due to the convenience sampling employed. The survey included more female participants, the level of educational attainment in our sample was higher, and urban dwellers were more represented in our sample than that of the population of Serbia. An alternative sampling strategy might have produced a more representative sample and enabled us to make stronger generalizations than envisioned by this exploratory study. The questionnaire was available only through the Internet, which excluded people without internet access. Further, the study was conducted only in Serbia, and included 337 participants. Filling out the questionnaire took a lot of time, which may have discouraged potential participants. Even so, certain aspects such as the differentiation of respondents to those prone to utilitarian reasoning and those more deontologically oriented required an even more extensive questionnaire, that this exploratory study was designed to cover.

## Conclusion

We have largely confirmed the findings of Specker et al. [1]. Our research showed that the respondents in our survey were less supportive of pharmacological than of non-pharmacological means of ME, that is, that the respondents largely disapproved of biomedical interventions for ME. The public in the US and in Serbia appears to generally eschew pharmacological MBE. The US population appears to be more open to non-biomedical means of attaining ME than the respondents in Serbia.

Although overall support for the idea of MBE is poor, there are some areas where people tend to be more receptive. For instance, the most important aspect of whether someone is going to be receptive or not to MBE is how close to home the measures hit, the fear of the means to be used, as well as reservations toward the idea of “identity change”. Respondents consider as less important aspects such as global wellbeing, security, and encroachment upon personal freedoms. Any attempt to sway the public (either way) should therefore be guided by these principles (at least in Serbia). Ethical concerns when it comes to trying to change public opinion on MBE constitute a separate debate. This study aims to inform the debate on bioethics, and its findings could be used for implementing or resisting the implementation of MBE related policies.

For example, it wouldn’t be as productive to offer arguments of global wellbeing if some policy regarding MBE is to be adopted, nor should a potential promoter of the idea concern herself with the idea of freedom (while this might be important conceptually, it matters little to the respondents compared to the other factors).

Although research of Specker et al. [1] does not include factor analysis, the hypotheses tested in their paper hint at the existence of the same factors. It is not only important to ascertain whether the public is receptive to MBE, but to understand the underlying reasons and motivations. Any future attempts to implement MBE policies should be informed by these considerations.

This research and the factors isolated could serve also as a basis for future research. Our research is one of the first studies that examine motivations behind people’s attitudes towards MBE. It also underlines the importance of factor analysis, as well as the importance of the factors we have isolated. Further studies on a larger and more representative sample, using a revised questionnaire (informed by our findings) are needed to confirm (or disconfirm) our analysis. A potential further breakdown of the isolated factors into sub-factors (that might be hiding beneath) may be possible and requires further investigation. Our research demonstrated that the respondents shared some of the opinions and concerns about MBE which have been present in philosophical discourse for some time now. A deeper understanding of the dimensions of the attitudes concerning MBE has some practical implications. While the subject is often framed by the extremes of a possible dystopian future, there are quite a few real-world examples of MBE that are in use right now. The acceptance, wider adoption, and welcoming of the new methods depend on the general support of the populace (or at least lack of active resistance to it).

## Supplementary Information

**Additional file 1**. Appendix.

**Additional file 2**. Code for statistical analysis.

## Data Availability

The raw survey data is in the figshare repository https://doi.org/10.6084/m9.figshare.14502297.v1 (anonymized link included in the Additional file 2: Code file).

## Abbreviations

MBE: Moral bioenhancement
ME: Moral enhancement
CE: Cognitive enhancement
MDMA: 3,4-Methylenedioxymethamphetamine, commonly known as ecstasy, a psychoactive drug
US: United States

## Footnotes

1 The research has been approved by The European Division of the UNESCO Chair in Bioethics and the Center for the Study of Bioethics in Belgrade.

2 In the second part of our research, which will be published as a separate paper, we investigate whether factors such as the difference between self and other, own child and other children, MBE of the children, voluntary/mandatory distinction, involvement in violence, and religion have an influence on people’s attitudes towards MBE. We have chosen this division in advance. The study encompassed two different and important topics that, due to the scope of the paper, could not be minutely analyzed in one paper.

3 See: Sparrow [24].

4 Four of them were modified versions from research by Specker et al. [1], and one of them was inspired by an example given by Savulescu and Persson [7].

5 For more on deontology see: [68]. For more on utilitarianism see: [69].

## References

[1] Specker J, Schermer MHN, Reiner PB (2017). Public attitudes towards moral enhancement. Evidence that means matter morally. Neuroethics.

[2] Knobe J, Nichols S. Experimental philosophy. In: Zalta EN, editor. The Stanford Encyclopedia of Philosophy [Internet]. Winter 2017. Metaphysics Research Lab, Stanford University; 2017. https://plato.stanford.edu/archives/win2017/entries/experimental-philosophy/

[3] Knobe JM, Nichols S (2008). Experimental philosophy.

[4] Doris JM, Greene JD, Griffiths PE, Harman G, Knobe J, Machery E, et al. Experimental Philosophy Defended (Leiter) [Internet]. Leiter reports: a philosophy blog. 2006 [cited 2021 Oct 25]. https://leiterreports.typepad.com/blog/2006/03/experimental_ph.html

[5] Douglas T (2008). Moral enhancement. J Appl Philos.

[6] Raus K, Focquaert F, Schermer M, Specker J, Sterckx S (2014). On Defining moral enhancement: a clarificatory taxonomy. Neuroethics.

[7] Savulescu J, Persson I (2012). Moral enhancement, freedom, and the god machine: Sugden SJB, editor. Monist.

[8] Baumgartner T, Heinrichs M, Vonlanthen A, Fischbacher U, Fehr E (2008). Oxytocin shapes the neural circuitry of trust and trust adaptation in humans. Neuron.

[9] Theodoridou A, Rowe AC, Penton-Voak IS, Rogers PJ (2009). Oxytocin and social perception: oxytocin increases perceived facial trustworthiness and attractiveness. Horm Behav.

[10] Hurlemann R, Patin A, Onur OA, Cohen MX, Baumgartner T, Metzler S (2010). Oxytocin enhances amygdala-dependent, socially reinforced learning and emotional empathy in humans. J Neurosci.

[11] Kosfeld M, Heinrichs M, Zak PJ, Fischbacher U, Fehr E (2005). Oxytocin increases trust in humans. Nature.

[12] Crockett MJ, Siegel JZ, Kurth-Nelson Z, Ousdal OT, Story G, Frieband C (2015). Dissociable effects of serotonin and dopamine on the valuation of harm in moral decision making. Curr Biol.

[13] Cohen Kadosh R, Soskic S, Iuculano T, Kanai R, Walsh V (2010). Modulating neuronal activity produces specific and long-lasting changes in numerical competence. Curr Biol.

[14] Carter AJ, Hall W, European Monitoring Centre for Drugs and Drug Addiction, editors. Addiction neurobiology: ethical and social implications. Luxembourg: Office for Official Publications of the European Communities; 2009. (EMCDDA monographs).

[15] Persson I, Savulescu J (2008). The perils of cognitive enhancement and the urgent imperative to enhance the moral character of humanity. J Appl Philos.

[16] Persson I, Savulescu J (2019). The duty to be morally enhanced. Topoi.

[17] Persson I, Savulescu J (2019). The evolution of moral progress and biomedical moral enhancement. Bioethics.

[18] Persson I, Savulescu J (2013). Getting moral enhancement right: the desirability of moral bioenhancement: getting moral enhancement right. Bioethics.

[19] Frank LE (2020). What do we have to lose? Offloading through moral technologies: moral struggle and progress. Sci Eng Ethics.

[20] Conan GM (2020). Frequently overlooked realistic moral bioenhancement interventions. J Med Ethics.

[21] Macpherson I, Roqué MV, Segarra I (2019). Moral enhancement, at the peak of pharmacology and at the limit of ethics. Bioethics.

[22] Azevedo MA (2016). The misfortunes of moral enhancement. JMPHIL.

[23] Balistreri M (2016). Hopes and limits of moral bioenhancement. Medicina & Storia.

[24] Sparrow R (2014). Better living through chemistry? A reply to savulescu and persson on ‘moral enhancement’: a reply to Savulescu and Persson on ‘moral enhancement’. J Appl Philos.

[25] Fitz NS, Nadler R, Manogaran P, Chong EWJ, Reiner PB (2014). Public attitudes toward cognitive enhancement. Neuroethics.

[26] Riis J, Simmons JP, Goodwin GP (2008). Preferences for enhancement pharmaceuticals: the reluctance to enhance fundamental traits. J Consum Res.

[27] Kahane G, Everett JAC, Earp BD, Caviola L, Faber NS, Crockett MJ (2018). Beyond sacrificial harm: a two-dimensional model of utilitarian psychology. Psychol Rev.

[28] R Core Team. R: A language and environment for statistical computing [Internet]. Vienna, Austria: R Foundation for Statistical Computing; 2014. http://www.R-project.org/

[29] Revelle WR. psych: procedures for personality and psychological research. 2017.

[30] Kassambara A. rstatix: pipe-friendly framework for basic statistical tests [Internet]. 2020. https://CRAN.R-project.org/package=rstatix

[31] Aragon TJ. epitools: epidemiology tools [Internet]. 2020. https://CRAN.R-project.org/package=epitools

[32] Budić M, Galjak M, Rakić V. SI_data.xlsx [Internet]. figshare; 2021 [cited 2021 Oct 14]. p. 256223 Bytes. https://figshare.com/articles/dataset/SI_data_xlsx/14502297/1

[33] Harman HH, Jones WH (1966). Factor analysis by minimizing residuals (minres). Psychometrika.

[34] Kaiser HF (1974). An index of factorial simplicity. Psychometrika.

[35] Cronbach LJ (1951). Coefficient alpha and the internal structure of tests. Psychometrika.

[36] Bandalos DL, Boehm-Kaufman MR. Four common misconceptions in exploratory factor analysis. In: Statistical and methodological myths and urban legends: doctrine, verity and fable in the organizational and social sciences. New York: Routledge/Taylor & Francis Group; 2009. p. 61–87.

[37] Tucker LR, Lewis C (1973). A reliability coefficient for maximum likelihood factor analysis. Psychometrika.

[38] Hu L, Bentler PM (1999). Cutoff criteria for fit indexes in covariance structure analysis: conventional criteria versus new alternatives. Struct Equ Modeling.

[39] Jackson JE. Oblimin Rotation. In: Armitage P, Colton T, editors. Encyclopedia of Biostatistics [Internet]. Chichester, UK: John Wiley & Sons, Ltd; 2005 [cited 2020 Sep 27]. p. b2a13060. 10.1002/0470011815.b2a13060

[40] Kelley K, Lai K (2011). Accuracy in parameter estimation for the root mean square error of approximation: sample size planning for narrow confidence intervals. Multivar Behav Res.

[41] Williams EF, Steffel M (2014). Double standards in the use of enhancing products by self and others. J Consum Res.

[42] Harris J (2011). Moral enhancement and freedom: moral enhancement and freedom. Bioethics.

[43] Fukuyama F. Our posthuman future: consequences of the biotechnological revolution. 1. Picador ed. New York: Picador; 2003.

[44] President’s Council on Bioethics (U.S.), Kass L, editors. Beyond therapy: biotechnology and the pursuit of happiness. 1st ed. New York: ReganBooks; 2003.

[45] Pugh J (2019). Moral bio-enhancement, freedom, value and the parity principle. Topoi.

[46] Sandel MJ (2007). The case against perfection: ethics in the age of genetic engineering.

[47] Simkulet W (2016). Intention and moral enhancement: intention and moral enhancement. Bioethics.

[48] Protopapadakis ED (2017). In defense of pharmaceutically enhancing human morality. Curr Ther Res.

[49] Walker M (2009). Enhancing genetic virtue: a project for twenty-first century humanity?. Politics Life Sci.

[50] Specker J, Focquaert F, Raus K, Sterckx S, Schermer M (2014). The ethical desirability of moral bioenhancement: a review of reasons. BMC Med Ethics.

[51] Crutchfield P (2020). It is better to be ignorant of our moral enhancement: a reply to Zambrano. Bioethics.

[52] Baccarini E, Malatesti L (2017). The moral bioenhancement of psychopaths. J Med Ethics.

[53] Sirgiovanni E, Garasic MD (2020). Commentary: the moral bioenhancement of psychopaths. Front Psychol.

[54] Rakić V, Ćirković M (2016). Confronting existential risks with voluntary moral bioenhancement. J Evol Technol.

[55] Agar N (2005). Liberal eugenics: in defence of human enhancement.

[56] Spence SA (2008). Can pharmacology help enhance human morality?. Br J Psychiatry.

[57] Hansen J, Wänke M (2009). Liking what’s familiar: the importance of unconscious familiarity in the mere-exposure effect. Soc Cogn.

[58] Imamoğlu Ç, Imamoğlu EO (2006). Relationship between familiarity, attitudes and preferences: assisted living facilities as compared to nursing homes. Soc Indic Res.

[59] Zajonc RB (2001). Mere exposure: a gateway to the subliminal. Curr Dir Psychol Sci.

[60] Zajonc RB (1968). Attitudinal effects of mere exposure. J Personal Soc Psychol.

[61] Knoke D, Isaac L (1976). Quality of higher education and sociopolitical attitudes. Soc Forces.

[62] Brennan J, Chanfreau J, Finnegan J, Griggs J, Kiss Z, Park A. The effect of higher education on graduates’ attitudes: secondary analysis of the british social attitudes survey [Internet]. Department for Business Innovation & Skills; 2015. (BIS research paper). https://books.google.rs/books?id=qOPWvQEACAAJ

[63] Bobo L, Licari FC (1989). Education and political tolerance: testing the effects of cognitive sophistication and target group affect. Public Opin Q.

[64] Hastie B (2007). Cold hearts and bleeding hearts: disciplinary differences in university students’ sociopolitical orientations. J Soc Psychol.

[65] Magolda MBB, Astin AW (1993). What “doesn’t” matter in college?. Educ Res.

[66] Verdier É. Baudelot Christian & Leclercq François (dir.), avec la collaboration de Armand Chatard, Boris Gorille & Elena Satchkova. Les effets de l’éducation : r: Paris : La Documentation française, 2005. 365 p. rfp. 2006;(156):171–2.

[67] Chatard A, Selimbegovic L (2007). The impact of higher education on egalitarian attitudes and values: contextual and cultural determinants. Social Pers Psych Compass.

[68] Alexander L, Moore M. Deontological Ethics. In: Zalta EN, editor. The Stanford Encyclopedia of Philosophy [Internet]. Winter 2016. Metaphysics Research Lab, Stanford University; 2016. https://plato.stanford.edu/archives/win2016/entries/ethics-deontological/

[69] Sinnott-Armstrong W. Consequentialism. In: Zalta EN, editor. The Stanford Encyclopedia of Philosophy [Internet]. Summer 2019. Metaphysics Research Lab, Stanford University; 2019. https://plato.stanford.edu/archives/sum2019/entries/consequentialism/

