# Supplementary material for: What drives public attitudes towards moral bioenhancement and why it matters: an exploratory study
Source: BMC Med Ethics. 2021 Dec 9;22:163. doi: 10.1186/s12910-021-00732-1 (PMC8656088; doi:10.1186/s12910-021-00732-1)
Supplement: Supplementary file 1 — Additional file 1. Appendix. [file 12910_2021_732_MOESM1_ESM.pdf]

What drives public attitudes towards moral bioenhancement and why it matters: an exploratory study

Marina Budić
Marko Galjak
Vojin Rakić

Text S1. The Questionnaire

General information

1. Sex

   Male
   Female
   Other

2. Year of birth
3. Municipality of residence (Not included in this study)
4. Country of residence (Not included in this study)
5. Type of residence
   Urban
   Rural
6. Level of education

   Primary school (lower grades: 4 years)
   Primary school (higher grades: 8 years in total)
   Secondary school
   Three-year professional school
   Four-year professional school or gymnasium
   Higher education
   A University degree
   Bachelor's degree
   Master's degree
   Doctoral degree

Moral Bioenhancement

Moral bioenhancement implies the improvement of moral propensities or motives that affect self-control, empathy, and other desirable characteristics that discourage tendencies toward violence and aggression. Although our moral conduct is complex, it is possible to distinguish several characteristics that have a biological and genetic basis and can be influenced by certain drugs. These are readiness to cooperate with other people, impulse control, altruism, and a sense of justice, and these characteristics further influence the development of empathy, solidarity, gratitude, and other desirable characteristics, as well as reducing the tendency towards violent and aggressive behavior.

Progress in neuroscience allows the use of drugs that affect aggression to reduce, propensity for empathy, cooperation, and trust. There are also potential non-pharmacological means such as various types of brain stimulation.

The example of bioenhancement: a pill that increases empathy or the sense of justice.

7. I'm familiar with this topic and have thought about this topic before.

not at all	1	2	3	4	5	6	7	completely

8. I think moral bioenhancement should be MANDATORY for criminals. (e.g., a pill that increases empathy and a sense of justice).
9. I think moral bioenhancement should be VOLUNTARY for criminals.
10. I believe moral bioenhancement should be MANDATORY for all the adults who manifest some form of immoral behavior.
11. I believe moral bioenhancement should be VOLUNTARY and allowed to anyone who wants to improve their character traits, motives, and behavior.
12. I believe moral bioenhancement should be MANDATORY for all the children who manifest some form of immoral behavior.
13. I believe moral bioenhancement should be VOLUNTARY and allowed to all the children whose parents want to improve the character traits, motives, and behavior of their children.
14. Moral bioenhancement would seriously jeopardize the identity of the person who undergoes this intervention.
15. Moral bioenhancement isn't correct because immoral means are used to achieve it.
16. Moral bioenhancers would seriously jeopardize/threaten and limit the freedom and autonomy of the person using it. This person wouldn't have the freedom to act the way they really wanted.
17. It would be better to use traditional methods of moral enhancement (such as education and upbringing) than methods of bioenhancement.
18. I am an opponent of the use of any form of pharmacological means and drugs, if there are natural or traditional methods for a particular problem, even if they are less effective.
19. I find there is no need for moral enhancement and that people nowadays are moral enough.

20. If a person is morally enhanced, it doesn't matter how did they achieve it, but the way they behave and the outcomes of their actions. The end sometimes, as in the case of moral behavior, justifies the means.
21. Scientific and technological progress allows cognitive enhancement, which would allow individuals or groups of people to cause damage and to harm others through the means of mass biological or nuclear destruction. Scientific progress and cognitive enhancement can be dangerous without moral enhancement.
22. In my opinion, moral education, i.e. upbringing, is sufficient, and that there is no need for using bioenhancers.
23. Humans are given certain characteristics and abilities by nature, and it should never be affected by technological means.
24. I believe some restrictions of freedom are justified. For example, the freedom of the prisoners is restricted, which is fair; also, it is okay to restrict freedom to a pedophile so as not to rape children. Thus, it is justified for people who tend towards immoral behavior to restrict freedom to do so to some extent, and to redirect their behavior.
25. No restriction of freedom is justified, because freedom is the highest value of humanity.
26. Moral bioenhancement could cause a change in the identity of the person undergoing bioenhancement interventions. This enhancement can alter a person's essential characteristics, which is very negative.
27. Even if moral bioenhancement leads to changes in a person's fundamental traits, in the case of negative traits such as, for example, impulsiveness, there is nothing wrong with reducing or modifying one's fundamental trait of impulsiveness.
28. Although the means of moral bioenhancement aren't natural, we also use other medicaments and medical interventions that are also unnatural. Therefore, moral bioenhancement shouldn't be rejected on the ground that it is unnatural.
29. Although the means and methods of moral bioenhancement may be characterized as unusual, it wouldn't be right to dismiss something simply because it is unusual for someone.
30. If people were allowed to increase their cognitive abilities (memory, attention, alertness), it wouldn't require moral bioenhancement too. Most people wouldn't have a propensity for immoral behavior if they improved their cognitive capacities.
31. It wouldn't be morally right to use moral bioenhancers.

Example 1

32. Please read the example and answer the questions below:

    (1) Philip is a professor of mathematics at the University of Belgrade. Every day, he passes a beggar sitting in the front of his college. The woman is in rags and asks for some money for shelter that night. Philip always averts his gaze and walks as far as possible away from her. He never gives her any money, although he is relatively wealthy. Philip takes a drug that makes him more interested in the suffering of others, more empathetic, more capable of imagining what it would be like to be in another person’s shoes. He looks at the beggar, reflects more about her suffering

and so decides to give the beggar food. He does not give money because he believes the beggar will use it imprudently. In this case, Philip is not only blindly generous but rationally thinks of the most appropriate way to help the beggar. Imagine that Philip, when he took the drug, always behaved in the morally correct way. He would not be unfree, but the most virtuous person.

To what degree do you think that it would be a good idea for Philip to use drugs that would make him more empathetic and inclined to help others?

not at all 1 2 3 4 5 6 7 completely

Please explain why you answered as you did. (optional question)

____

To what degree would you be willing to participate in the program yourself?

Please explain why you answered as you did. (optional question)

2

Example 2

33. Please read the example and answer the questions below:

(2) Imagine that YOUR CHILD is a VICTIM of peer violence. Every day, your child suffers physical abuse, while on social media, violence continues through belittling, insulting, and telling lies about your child. The school has a program that has been shown to be effective in reducing peer violence in carefully carried out studies. The program involves the following: over the course of 4 weeks, each day the bully takes a PILL that increases empathy for others. The pill is based on the natural hormone oxytocin and improves the bully's ability to understand what other people are feeling. Several studies have shown that the program significantly reduces peer violence without any negative side effects. The effects of the pill are visible for several months after the program is complete.

To what degree do you think that it would be a GOOD IDEA for the bully to participate in a program like the one described above?

not at all 1 2 3 4 5 6 7 completely

Please explain why you answered as you did. (optional question)

____

To what degree do you think that it would be a good idea for the BULLY to be REQUIRED to participate in the program like the one described above?

Please explain why you answered as you did. (optional question)

If there was a reliable test that identified children who are at higher risk of being bullies in the future, to what degree do you think that it would be a good idea if they would be REQUIRED to participate in the program?

Please explain why you answered as you did. (optional question)

Given that the program increases empathy, to what degree do you think it would be a good idea for ALL CHILDREN (not just bullies or potential bullies) to be REQUIRED to participate in the program?

Please explain why you answered as you did. (optional question)

If this program increased empathy in everyone, to what degree would you think that society would be better off if EVERYONE was REQUIRED to participate in the program?

Please explain why you answered as you did. (optional question)

To what degree would you be willing to participate in the program yourself?

Please explain why you answered as you did. (optional question)

To what degree would you be willing to participate in the program yourself if it was clinically determined beyond any doubt that you have a lack of empathy?

Please explain why you answered as you did. (optional question)

Example 3

34. Please read the example and answer the questions below:

(3) Imagine that YOUR CHILD PARTICIPATES in peer violence and BULLIES other children. Every day, your child physically bullies and on social media belittles, insults, and tells lies about the other child. The school has a program that has been shown to be effective in reducing peer violence in carefully carried out studies. The program involves the following: over the course of 4 weeks, each day your child takes a PILL that increases empathy for others. The pill is based on the natural hormone oxytocin and improves the bully's ability to understand what other people are feeling. Several studies have shown that the program significantly reduces peer violence without any negative side effects. The effects of the pill are visible for several months after the program is complete.

To what degree do you think that it would be a GOOD IDEA that IF YOU WANT your child to participate in a program like the one described above?

Please explain why you answered as you did. (optional question)

To what degree do you think that it would be a GOOD IDEA for your child to be REQUIRED to participate in a program like the one described above?

Please explain why you answered as you did. (optional question)

Example 4

35. Please read the example and answer the questions below:

(4) Imagine that YOUR CHILD is a VICTIM of peer violence. Every day, your child suffers physical abuse, while on social media, violence continues through belittling, insulting, and telling lies about your child. The school has a program that has been shown to be effective in reducing peer violence in carefully carried out studies. The program involves the following: over the course of 4 weeks, each day the bully plays a VIDEO GAME that increases empathy for others. The video game is based on best educational practices and improves the bully's ability to understand what other people are feeling. Several studies have shown that the program significantly reduces peer violence without any negative side effects. The effects of the program are visible for several months after the program is complete.

To what degree do you think that it would be a GOOD IDEA for the BULLY to participate in a program like the one described above if he wants?

Please explain why you answered as you did. (optional question)

To what degree do you think that it would be a good idea for the bully to be REQUIRED to participate in a program like the one described above?

Please explain why you answered as you did. (optional question)

If there was a reliable test that identified children who are at higher risk of being bullies in the future, to what degree do you think that it would be a good idea if they would be REQUIRED to participate in the program?

Please explain why you answered as you did. (optional question)

Given that the program increases empathy, to what degree do you think it would be a good idea for ALL CHILDREN (not just bullies or potential bullies) to be REQUIRED to participate in the program?

Please explain why you answered as you did. (optional question)

If this program increased empathy in everyone, to what degree would you think that society would be better off if EVERYONE was REQUIRED to participate in the program?

Please explain why you answered as you did. (optional question)

To what degree would you be willing to participate in the program yourself?

Please explain why you answered as you did. (optional question)

To what degree would you be willing to participate in the program yourself if it was clinically determined beyond any doubt that you have a lack of empathy?

Please explain why you answered as you did. (optional question)

Example 5

36. Please read the example and answer the questions below:

(5) Imagine that YOUR CHILD PARTICIPATES in peer violence and BULLIES other children. Every day, your child physically bullies and on the social media belittles, insults, and tells lies about the other child. The school has a program that has been shown to be effective in reducing peer violence in carefully carried out studies. The program involves the following: over the course of 4 weeks, each day the bully plays a VIDEO GAME that increases empathy for others. The video game is based on best educational practices, and improves the bully's ability to understand what other people are feeling. Several studies have shown that the program significantly reduces peer violence without any negative side effects. The effects of the program are visible for several months after the program is complete.

To what degree do you think that it would be a GOOD IDEA that IF YOU WANT your child to participate in a program like the one described above?

Please explain why you answered as you did. (optional question)

To what degree do you think that it would be a GOOD IDEA for your child to be REQUIRED to participate in a program like the one described above?

Please explain why you answered as you did. (optional question)

Final questions

37. Have you ever been a victim of violence, violent behavior, or bullying? (Not included in this study)

    Yes
    No
    I don't want to answer.

38. Have you ever bullied another or violently treated someone (even though you may have had realized it many years later)? (Not included in this study)

    Yes
    No
    I don't want to answer.

39. What religion do you belong to? (Not included in this study)

I'm not religious.

Christianity — Orthodox

Christianity — Catholic

Christianity — Protestant

Islam

Buddhism

Other

40. If you have picked a religion in the previous question, to what extent would you rate your own religiousness? (Not included in this study)

41. To what degree do you think it is more important to nurture individualism and freedom as opposed to state authority and collectivism? (Not included in this study)

42. To what degree do you consider it is important to nurture the traditional values and culture that you belong to? (Not included in this study)

43. To what degree do you agree with the following statements:

The end justifies the means.

not at all	1	2	3	4	5	6	7	completely

It is permissible to torture an innocent person if it will lead to information that will prevent a terrorist attack that would kill hundreds of people.

It is immoral to lie to a person, even though it is for their own good and in the end, it will really have a positive effect on them.

There are some things and actions that are simply good or bad, regardless of their consequences.

Criminals should be punished because they deserve it, even though it doesn't protect the public nor deter others from committing crimes in the future.

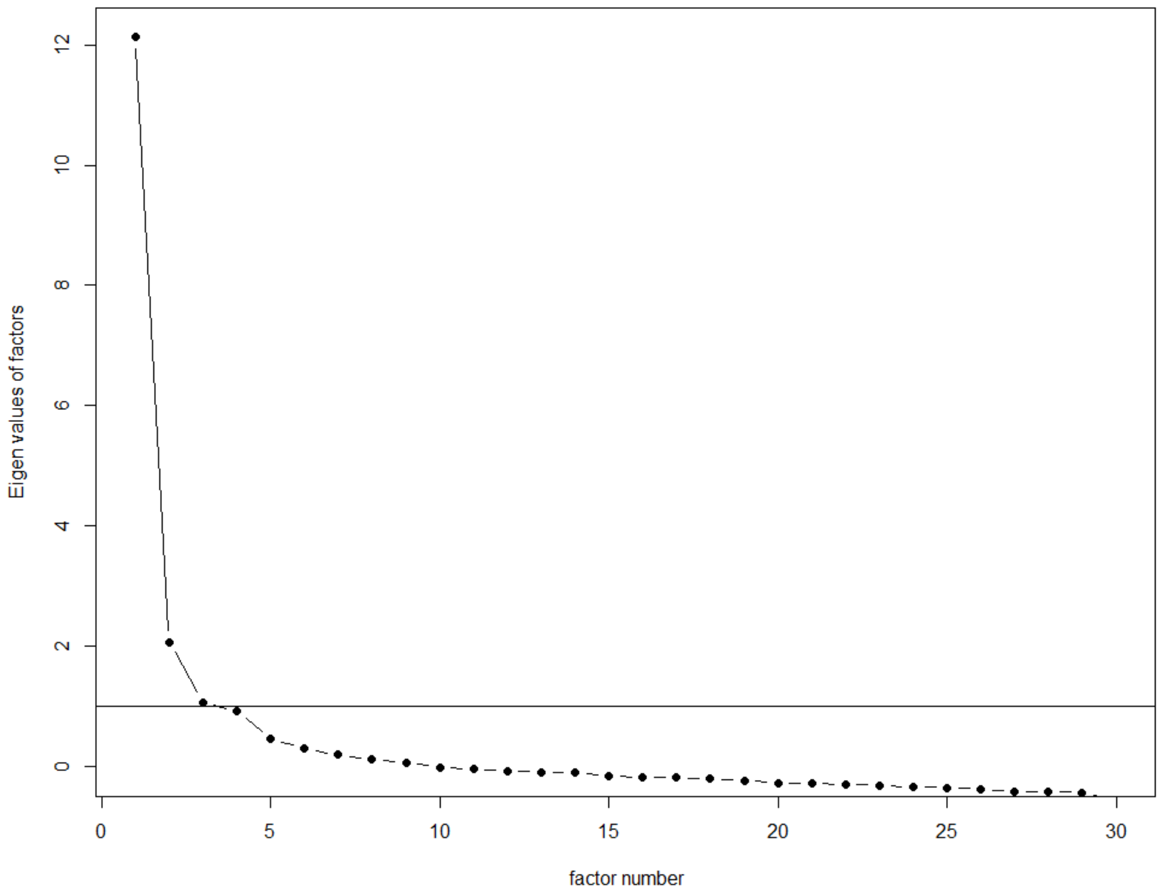

Figure S1 Scree plot

Table S1. Factor loadings of each question

No.	Q.	Abbreviated Questions	General - Closeness	Fear of Change	Mandatory Security	Voluntariness
1	8	MB should be MANDATORY for criminals. (e.g., a pill that increases empathy and a sense of justice).	0.4	-0.15	0.41	0.04
2	9	MB should be VOLUNTARY for criminals.	-0.06	0.23	-0.24	0.56
3	10	MB should be MANDATORY for all the adults who manifest some form of immoral behavior.	0.24	-0.15	0.66	0.05
4	11	MB should be VOLUNTARY and allowed to everyone anyone who wants to improve their character traits, motives,	0.06	-0.04	0.03	0.73
5	12	MB should be MANDATORY for all the children who manifest some form of immoral behavior.	0.35	-0.13	0.54	0.09
6	13	MB should be VOLUNTARY and allowed to all the children whose parents want to improve the character traits,	0.05	-0.12	0.36	0.49
7	14	MB would seriously jeopardize the identity of the person who undergoes this intervention.	0.01	0.54	-0.29	-0.12
8	15	MB isn't correct because immoral means are used to achieve it.	0.03	0.77	-0.04	-0.05
9	16	MB would seriously jeopardize/threaten and limit the freedom and autonomy of the person using it. This person would	0.05	0.73	-0.24	-0.01
10	17	It would be better to use traditional methods of ME (such as education and upbringing) than methods of bioenhancement	-0.03	0.65	-0.2	0.13
11	18	I am an opponent of pharmacological means if there are natural or traditional methods for a particular problem (even if	-0.09	0.61	0.2	0.15
12	20	If morally enhanced, it doesn't matter how it's achieved, but the way it affects behaves and the outcomes of their actions.	0.41	-0.18	0.19	0.12
13	22	In my opinion, moral education, i.e. upbringing, is sufficient, and that there is no need for using bioenhancers.	-0.18	0.57	-0.12	0.11
14	23	Humans are given certain characteristics and abilities by nature, and it should never be affected by technological means.	-0.03	0.68	0.15	-0.01
15	26	MB could cause a change in the identity of the person undergoing bioenhancement interventions. It can alter a person's essential characteristics, which is very negative.	-0.02	0.7	-0.07	-0.05
16	27	Even if MB changes a person's fundamental traits, in the case of negative traits (e.g., impulsiveness), there is nothing	0.36	-0.27	0.03	0.15
17	28	Means of MB aren't natural, but we use other drugs and medical interventions that are also unnatural. MB shouldn't be	0.19	-0.51	-0.2	0.21
18	29	Although the means and methods of MB may be characterized as unusual, it wouldn't be right to dismiss something	0.29	-0.22	-0.29	0.25
19	31	It wouldn't be morally right to use moral bioenhancers.	-0.07	0.7	-0.03	-0.1
20	32	To what degree do you think that it would be a good idea for Philip to use drugs that would make him more empathetic	0.56	-0.18	-0.03	0.12
21	32	To what degree do you think that it would be a good idea for you to use drugs that would make you more empathetic	0.73	-0.21	-0.24	-0.12
22	33	Would it be a good idea for the bully to take the pill?	0.63	-0.11	0.06	0.2
23	33	Would it be a good idea to make it mandatory for the bully to take the pill?	0.7	0	0.26	0.03
24	33	Would it be a good idea to make it mandatory for all the potential bullies to take the pill?	0.76	0.12	0.27	-0.01
25	33	Would it be a good idea for the pill to be mandatory for all children?	0.8	0.16	0.19	-0.16
26	33	Would it be a good idea for everyone to take the pill?	0.77	0.13	0.14	-0.05
27	33	To what degree would you be willing to take the pill?	0.89	-0.02	-0.21	-0.07
28	33	To what degree would you be willing to take the pill, if it were clinically proven that you lack empathy?	0.78	-0.13	-0.18	0.12
29	33	Would you like your child to take the pill if they are a bully?	0.77	-0.09	-0.01	0.11
30	33	Would you like your child to be mandated to take the pill?	0.84	-0.01	0.15	-0.03

No. refers to the number of variables in Figure 1 of the main text.

Q. – refers to the number of question or scenario given in the Text S1 of the Appendix

Table S2. List of questions used for composite scores

Q.	Abbreviated Questions	Pharm. means	Non-Pharm. means	Total MBE Sup.	Utilitarianism
8	MB should be MANDATORY for criminals. (e.g., a pill that increases empathy and a sense of justice).			0.69	
9	MB should be VOLUNTARY for criminals.			-0.04	
10	MB should be MANDATORY for all the adults who manifest some form of immoral behavior.			0.69	
11	MB should be VOLUNTARY and allowed to everyone anyone who wants to improve their character traits, motives, and behavior.			0.46	
12	MB should be MANDATORY for all the children who manifest some form of immoral behavior.			0.76	
13	MB should be VOLUNTARY and allowed to all the children whose parents want to improve the character traits, motives, and behavior			0.54	
14	MB would seriously jeopardize the identity of the person who undergoes this intervention.			0.55	
15	MB isn't correct because immoral means are used to achieve it.			0.49	
16	MB would seriously jeopardize/threaten and limit the freedom and autonomy of the person using it. This person would lose the freedom			0.55	
17	It would be better to use traditional methods of ME (such as education and upbringing) than methods of bioenhancement			-0.47	
18	I am an opponent of pharmacological means if there are natural or traditional methods for a particular problem (even if less effective).			-0.27	
20	If morally enhanced, it doesn't matter how it's achieved, but the way it affects behaves and the outcomes of their actions.			-0.65	
22	In my opinion, moral education, i.e. upbringing, is sufficient, and that there is no need for using bioenhancers.			0.52	
23	Humans are given certain characteristics and abilities by nature, and it should never be affected by technological means.			-0.37	
25	No restriction of freedom is justified because freedom is the highest value of humanity.			-0.22	
26	MB could cause a change in the identity of the person undergoing bioenhancement interventions. It can alter a person's essential characteristics, which is very negative.			0.52	
27	Even if MB changes a person's fundamental traits, in the case of negative traits (e.g., impulsiveness), there is nothing wrong with reducing/removing them.			0.61	
28	Means of MB aren't natural, but we use other drugs and medical interventions that are also unnatural. MB shouldn't be rejected on the ground that it is unnatural.			0.48	
29	Although the means and methods of MB may be characterized as unusual, it wouldn't be right to dismiss something simply because it is unusual for someone.			0.39	
31	It wouldn't be morally right to use moral bioenhancers.			-0.56	
33	Would it be a good idea for the bully to take the pill?	0.74		0.78	
33	Would it be a good idea to make it mandatory for the bully to take the pill?	0.81		0.80	
33	Would it be a good idea to make it mandatory for all the potential bullies to take the pill?	0.82		0.79	
33	Would it be a good idea for the pill to be mandatory for all children?	0.78		0.67	
33	Would it be a good idea for everyone to take the pill?	0.78		0.70	
33	To what degree would you be willing to take the pill?	0.78		0.71	
33	To what degree would you be willing to take the pill, if it were clinically proven that you lack empathy?	0.77		0.77	
34	Would it be a good idea for the bully to play the video game?		0.67		
34	Would it be a good idea to make it mandatory for the bully to play the video game?		0.83		
34	Would it be a good idea to make it mandatory for all the potential bullies to play the video game?		0.81		
34	Would it be a good idea for the video game should be mandatory for all children?		0.85		
34	Would it be a good idea for everyone to play the video game?		0.86		
34	To what degree would you be willing to play the video game?		0.84		
34	To what degree would you be willing to play the video game, if it were clinically proven that you lack empathy?		0.80		
43	The end justifies the means.				0.27
43	It is permissible to torture an innocent person if it will lead to information that will prevent a terrorist attack that would kill hundreds of people.				0.34
43	It is immoral to lie to a person, even though it is for their own good and in the end, it will really have a positive effect on them.				0.29
43	There are some things and actions that are simply good or bad, regardless of their consequences.				0.40
43	Criminals should be punished because they deserve it, even though it doesn't protect the public nor deter others from committing crimes in the future.				0.31
