# Supplementary material for: What drives public attitudes towards moral bioenhancement and why it matters: an exploratory study
Source: BMC Med Ethics. 2021 Dec 9;22:163. doi: 10.1186/s12910-021-00732-1 (PMC8656088; doi:10.1186/s12910-021-00732-1)
Supplement: Supplementary file 2 — Additional file 2. Code for statistical analysis. [file 12910_2021_732_MOESM2_ESM.pdf]

What drives public attitudes towards moral bioenhancement and why it matters: an exploratory study

Marina Budić, Marko Galjak, Vojin Rakić

10/26/2021

Supplementary material

This is an R Markdown document with code needed to run the statistical analysis for the article this material supplements.

The Libraries

We begin by loading the required libraries

```
require(openxlsx)
require(psych)
require(GPArotation)
require(dplyr)
require(FactoMineR)
require(ggplot2)
require(ggcorrplot)
require(rstatix)
require(ggthemes)
require(epitools)
```

Helper functions

Creating the helper functions which we will need

```
# fa.CFI() function credited to github user @tonosan
# https://gist.github.com/tonosan/cb7581f3459ae7c4217a

fa.CFI<-function(x){
  nombre<-paste(x,"CFI",sep = ".")
  nombre<-
    ((x$null.chisq-x$null.dof)-(x$STATISTIC-x$dof))/(x$null.chisq-x$null.dof)
  return(nombre)
}

#function that will help us reverse some Likert questions

reverse_likert <- function(x) {
```

```
  ifelse(x == 1, 7, ifelse(x == 2, 6, ifelse(x == 3, 5, ifelse(
    x == 5, 3, ifelse(x == 6, 2, ifelse(x == 7, 1, x))
  ))))
}
```

Loading the data

We are pulling the data from the online repository they have been uploaded to.

```
#we use the direct data link from the repository with doi: ANONYMIZED
url <-
  "https://ndownloader.figshare.com/files/27780570?private_link=28527d0c06302bfb6fa1"

survey_data_clean <- read.xlsx(url, check.names = T)
```

Cleaning the data

We are performing some cleaning and recoding of the data

```
#Coding Sex
survey_data_clean$X1..Pol <-
  factor(survey_data_clean$X1..Pol , levels = c("Muški", "Ženski"))
survey_data_clean$X1..Pol <- as.numeric(survey_data_clean$X1..Pol)

#Coding Urban/Rural
survey_data_clean$X5..Tip.naselja  <-
  factor(survey_data_clean$X5..Tip.naselja ,
         levels = c("Urbano", "Rurarlno"))
survey_data_clean$X5..Tip.naselja <-
  as.numeric(survey_data_clean$X5..Tip.naselja)

#Coding Familiarity with the term
survey_data_clean$X7_FAMILIARITY <-
  factor(survey_data_clean$X7_FAMILIARITY , levels = c("Da", "Ne"))
survey_data_clean$X7_FAMILIARITY <-
  as.numeric(survey_data_clean$X7_FAMILIARITY)

survey_data_clean <- survey_data_clean %>% select_if(is.numeric)
survey_data_clean$Timestamp <- NULL

#Calculating age based on the year of birth and year when the survey has been conducted
survey_data_clean$X2AGE <- 2020 - survey_data_clean$X2.Godina.rođenja
survey_data_clean$X2.Godina.rođenja <- NULL

#Removing one respondent due to incomplete answer
survey_data_clean <- survey_data_clean[-320, ]


#Reversing the negative Likert scale questions
survey_data_clean$X15_IDENTITY_P <-
  reverse_likert(survey_data_clean$X15_IDENTITY_N)
survey_data_clean$X17_MEANS_P <-
```

```
  reverse_likert(survey_data_clean$X17_MEANS_N)
survey_data_clean$X18_FREEDOM_P <-
  reverse_likert(survey_data_clean$X18_FREEDOM_N)
survey_data_clean$X22_MEANS_P <-
  reverse_likert(survey_data_clean$X22_MEANS_N)
survey_data_clean$X24_NEED_P <-
  reverse_likert(survey_data_clean$X24_NEED_N)
survey_data_clean$X26_FREEDOM_P <-
  reverse_likert(survey_data_clean$X26_FREEDOM_N)
survey_data_clean$X28_IDENTITY_P <-
  reverse_likert(survey_data_clean$X28_IDENTITY_N)
survey_data_clean$X31_NATURAL_P <-
  reverse_likert(survey_data_clean$X31_NATURAL_N)
survey_data_clean$X33_MEANS_P <- survey_data_clean$X33_MEANS_N
```

Descriptive statistics

```
#Sex
survey_data_clean$X1..Pol %>% table #/nrow(survey_data_clean)*100
```

```
## .
##   1   2
## 102 235
```

```
#Type of settlement they live in (urban/rural)
survey_data_clean$X5..Tip.naselja %>% table/nrow(survey_data_clean)*100
```

```
## .
##        1        2
## 87.24036 12.75964
```

```
#Level of education
survey_data_clean$Univeristy_degree %>% table
```

```
## < table of extent 0 >
```

```
survey_data_clean$AGE_BINS[survey_data_clean$X2AGE >= 25 & survey_data_clean$X2AGE < 30] <- "25-29"
survey_data_clean$AGE_BINS[survey_data_clean$X2AGE >= 30 & survey_data_clean$X2AGE < 35] <- "30-34"
survey_data_clean$AGE_BINS[survey_data_clean$X2AGE <25] <- "<25"
survey_data_clean$AGE_BINS[survey_data_clean$X2AGE >=35] <- ">35"
survey_data_clean$AGE_BINS <- factor(survey_data_clean$AGE_BINS , levels=c("<25","25-29","30-34",">35"))
survey_data_clean$AGE_BINS %>% table()
```

```
## .
##   <25 25-29 30-34   >35
##    58    78    70   131
```

```
survey_data_clean$X2AGE %>% mean
```

```
## [1] 35.08012
```

The factor analysis

We are pulling the data from the online repository they have been uploaded to.

```
#We select only variables that are directly related to moral bioenhancment
mbe_vars <-
  survey_data_clean %>% select(
    X9_MANDATORY_P_CRIM:X44_YOUR.CHILD_MANDATORY_P,-X21_NEED_N,
    -X16_NEED_P,
    -X26_FREEDOM_N,
    -X27_FREEDOM_P,
    -X23_RISK.OF.COGNITIVE_P,
    -X32_RISK.OF.COGNITIVE_N
  ) %>% colnames

#We pick the reverse Likert scales for the variables to be pointing in the same
#direction for calculating the Cronbach's alpha
mbe_vars_pos <-
  survey_data_clean %>% select(
    X9_MANDATORY_P_CRIM:X44_YOUR.CHILD_MANDATORY_P,
    -X21_NEED_N,
    -X16_NEED_P,
    -X26_FREEDOM_N,
    -X27_FREEDOM_P,-X23_RISK.OF.COGNITIVE_P,
    -X32_RISK.OF.COGNITIVE_N,
    -X15_IDENTITY_N,
    X15_IDENTITY_P,
    -X17_MEANS_N,
    X17_MEANS_P,
    -X18_FREEDOM_N,
    X18_FREEDOM_P,-X22_MEANS_N,
    X22_MEANS_P,
    -X24_NEED_N,
    X24_NEED_P,
    -X28_IDENTITY_N,
    X28_IDENTITY_P,
    -X31_NATURAL_N,
    X31_NATURAL_P
  ) %>% colnames

#Calculating The Kaiser-Meyer-Olkin factor adequacy measure.
KMO(survey_data_clean %>% select(mbe_vars))
```

```
## Note: Using an external vector in selections is ambiguous.
## i Use `all_of(mbe_vars)` instead of `mbe_vars` to silence this message.
## i See <https://tidyselect.r-lib.org/reference/faq-external-vector.html>.
## This message is displayed once per session.
```

```
## Kaiser-Meyer-Olkin factor adequacy
## Call: KMO(r = survey_data_clean %>% select(mbe_vars))
## Overall MSA =  0.93
## MSA for each item =
##          X9_MANDATORY_P_CRIM         X10_VOLUNTARY_P_CRIM
```

```
##                        0.94                         0.62
##       X11_MANDATORY_P_ADULTS       X12_VOLUNTARY_P_ADULTS
##                        0.91                         0.78
##     X13_MANDATORY_P_CHILDREN     X14_VOLUNTARY_P_CHILDREN
##                        0.92                         0.85
##               X15_IDENTITY_N                  X17_MEANS_N
##                        0.97                         0.95
##                X18_FREEDOM_N           X19_TRADITIONAL_P
##                        0.95                         0.92
##    X20_NATURAL_TRADITIONAL_P                  X22_MEANS_N
##                        0.93                         0.97
##                   X24_NEED_N                X25_NATURAL_P
##                        0.96                         0.94
##               X28_IDENTITY_N              X29_IDENTITY_P
##                        0.95                         0.96
##                X30_NATURAL_N                X31_NATURAL_N
##                        0.93                         0.92
##                  X33_MEANS_N                X34_GENERAL_P
##                        0.95                         0.95
##               X35_PERSONAL_P               X36_.BULLY_P
##                        0.92                         0.94
##        X37_BULLY_MANDATORY_P   X38_PREVENTION_MANDATORY_P
##                        0.94                         0.96
## X39_ALL.CHILDREN_MANDATORY_P    X40_EVERYBODY_MANDATORY_P
##                        0.90                         0.92
##               X41_PERSONAL_P     X42_PERSONAL_OBJECTIVE_P
##                        0.93                         0.96
##   X43_YOUR.CHILD_VOLUNTARY_P   X44_YOUR.CHILD_MANDATORY_P
##                        0.95                         0.95
```

```
#Confirming the internal consistency with Cronbach's alpha.

psych::alpha(survey_data_clean %>% select(mbe_vars_pos))
```

```
## Note: Using an external vector in selections is ambiguous.
## i Use `all_of(mbe_vars_pos)` instead of `mbe_vars_pos` to silence this message.
## i See <https://tidyselect.r-lib.org/reference/faq-external-vector.html>.
## This message is displayed once per session.

## Some items ( X10_VOLUNTARY_P_CRIM X19_TRADITIONAL_P X20_NATURAL_TRADITIONAL_P X25_NATURAL_P X33_MEANS
## probably should be reversed.
## To do this, run the function again with the 'check.keys=TRUE' option

##
## Reliability analysis
## Call: psych::alpha(x = survey_data_clean %>% select(mbe_vars_pos))
##
##   raw_alpha std.alpha G6(smc) average_r S/N    ase mean   sd median_r
##       0.86      0.84    0.93      0.15 5.4 0.0088  3.6 0.87     0.31
##
##  lower alpha upper     95% confidence boundaries
## 0.84 0.86 0.87
##
```

```
##  Reliability if an item is dropped:
##                             raw_alpha std.alpha G6(smc) average_r S/N alpha se
## X9_MANDATORY_P_CRIM              0.84      0.83    0.92      0.14 4.9   0.0096
## X10_VOLUNTARY_P_CRIM             0.87      0.85    0.93      0.17 5.8   0.0081
## X11_MANDATORY_P_ADULTS           0.84      0.83    0.92      0.15 4.9   0.0095
## X12_VOLUNTARY_P_ADULTS           0.85      0.84    0.92      0.15 5.2   0.0089
## X13_MANDATORY_P_CHILDREN         0.84      0.83    0.92      0.14 4.9   0.0096
## X14_VOLUNTARY_P_CHILDREN         0.85      0.84    0.92      0.15 5.1   0.0092
## X19_TRADITIONAL_P                0.87      0.86    0.94      0.18 6.4   0.0079
## X20_NATURAL_TRADITIONAL_P        0.87      0.86    0.93      0.17 6.1   0.0077
## X25_NATURAL_P                    0.87      0.86    0.93      0.18 6.2   0.0079
## X29_IDENTITY_P                   0.85      0.83    0.92      0.15 5.0   0.0093
## X30_NATURAL_N                    0.85      0.84    0.93      0.15 5.2   0.0091
## X33_MEANS_N                      0.88      0.87    0.94      0.18 6.5   0.0077
## X34_GENERAL_P                    0.84      0.83    0.92      0.14 4.9   0.0095
## X35_PERSONAL_P                   0.85      0.83    0.92      0.15 5.0   0.0095
## X36_.BULLY_P                     0.84      0.83    0.92      0.14 4.8   0.0098
## X37_BULLY_MANDATORY_P            0.84      0.83    0.92      0.14 4.8   0.0098
## X38_PREVENTION_MANDATORY_P       0.84      0.83    0.92      0.14 4.8   0.0097
## X39_ALL.CHILDREN_MANDATORY_P     0.85      0.83    0.92      0.15 4.9   0.0094
## X40_EVERYBODY_MANDATORY_P        0.84      0.83    0.92      0.14 4.9   0.0095
## X41_PERSONAL_P                   0.84      0.83    0.92      0.14 4.9   0.0096
## X42_PERSONAL_OBJECTIVE_P         0.84      0.83    0.92      0.14 4.8   0.0098
## X43_YOUR.CHILD_VOLUNTARY_P       0.84      0.83    0.92      0.14 4.8   0.0100
## X44_YOUR.CHILD_MANDATORY_P       0.84      0.83    0.92      0.14 4.7   0.0100
## X15_IDENTITY_P                   0.85      0.83    0.93      0.15 5.0   0.0093
## X17_MEANS_P                      0.85      0.84    0.92      0.15 5.1   0.0092
## X18_FREEDOM_P                    0.85      0.83    0.92      0.15 5.0   0.0093
## X22_MEANS_P                      0.88      0.87    0.94      0.18 6.6   0.0077
## X24_NEED_P                       0.85      0.84    0.93      0.15 5.1   0.0093
## X28_IDENTITY_P                   0.85      0.84    0.92      0.15 5.1   0.0092
## X31_NATURAL_P                    0.87      0.86    0.94      0.18 6.2   0.0080
##                             var.r med.r
## X9_MANDATORY_P_CRIM          0.15  0.30
## X10_VOLUNTARY_P_CRIM         0.16  0.33
## X11_MANDATORY_P_ADULTS       0.15  0.30
## X12_VOLUNTARY_P_ADULTS       0.16  0.33
## X13_MANDATORY_P_CHILDREN     0.15  0.30
## X14_VOLUNTARY_P_CHILDREN     0.16  0.31
## X19_TRADITIONAL_P            0.14  0.33
## X20_NATURAL_TRADITIONAL_P    0.15  0.33
## X25_NATURAL_P                0.14  0.33
## X29_IDENTITY_P               0.15  0.30
## X30_NATURAL_N                0.15  0.31
## X33_MEANS_N                  0.14  0.32
## X34_GENERAL_P                0.15  0.30
## X35_PERSONAL_P               0.15  0.30
## X36_.BULLY_P                 0.15  0.30
## X37_BULLY_MANDATORY_P        0.15  0.30
## X38_PREVENTION_MANDATORY_P   0.15  0.30
## X39_ALL.CHILDREN_MANDATORY_P  0.15  0.31
## X40_EVERYBODY_MANDATORY_P    0.15  0.30
## X41_PERSONAL_P               0.15  0.30
## X42_PERSONAL_OBJECTIVE_P     0.15  0.30
```

```
## X43_YOUR.CHILD_VOLUNTARY_P    0.15  0.30
## X44_YOUR.CHILD_MANDATORY_P    0.14  0.30
## X15_IDENTITY_P                0.15  0.30
## X17_MEANS_P                   0.15  0.30
## X18_FREEDOM_P                 0.15  0.30
## X22_MEANS_P                   0.14  0.33
## X24_NEED_P                    0.15  0.30
## X28_IDENTITY_P                0.15  0.30
## X31_NATURAL_P                 0.15  0.33
##
##  Item statistics
##                                 n  raw.r std.r  r.cor r.drop mean  sd
## X9_MANDATORY_P_CRIM           337  0.697  0.69  0.689   0.65  3.9 2.1
## X10_VOLUNTARY_P_CRIM          337 -0.026 -0.03 -0.084  -0.11  4.3 2.1
## X11_MANDATORY_P_ADULTS        337  0.688  0.69  0.689   0.64  3.2 2.0
## X12_VOLUNTARY_P_ADULTS        337  0.445  0.44  0.421   0.38  5.2 1.9
## X13_MANDATORY_P_CHILDREN      337  0.744  0.74  0.746   0.70  2.8 2.1
## X14_VOLUNTARY_P_CHILDREN      337  0.553  0.55  0.534   0.49  3.6 2.2
## X19_TRADITIONAL_P             337 -0.440 -0.44 -0.489  -0.49  5.3 1.7
## X20_NATURAL_TRADITIONAL_P     337 -0.228 -0.23 -0.284  -0.30  4.1 2.1
## X25_NATURAL_P                 337 -0.338 -0.33 -0.387  -0.40  3.6 1.8
## X29_IDENTITY_P                337  0.614  0.62  0.602   0.57  3.9 1.7
## X30_NATURAL_N                 337  0.476  0.48  0.457   0.42  4.7 1.9
## X33_MEANS_N                   337 -0.523 -0.52 -0.571  -0.57  3.8 1.8
## X34_GENERAL_P                 337  0.706  0.71  0.699   0.67  3.4 1.9
## X35_PERSONAL_P                337  0.655  0.65  0.655   0.61  2.5 1.9
## X36_.BULLY_P                  337  0.782  0.77  0.782   0.75  3.9 2.2
## X37_BULLY_MANDATORY_P         337  0.799  0.79  0.804   0.77  3.2 2.2
## X38_PREVENTION_MANDATORY_P    337  0.779  0.78  0.783   0.75  3.0 2.1
## X39_ALL.CHILDREN_MANDATORY_P  337  0.677  0.68  0.685   0.64  2.1 1.7
## X40_EVERYBODY_MANDATORY_P     337  0.697  0.70  0.701   0.66  2.5 1.9
## X41_PERSONAL_P                337  0.727  0.73  0.733   0.69  2.7 2.1
## X42_PERSONAL_OBJECTIVE_P      337  0.773  0.77  0.775   0.73  3.5 2.3
## X43_YOUR.CHILD_VOLUNTARY_P    337  0.816  0.81  0.820   0.78  3.5 2.3
## X44_YOUR.CHILD_MANDATORY_P    337  0.846  0.84  0.857   0.82  2.8 2.2
## X15_IDENTITY_P                337  0.580  0.59  0.567   0.53  3.7 1.9
## X17_MEANS_P                   337  0.510  0.51  0.499   0.45  4.6 1.9
## X18_FREEDOM_P                 337  0.575  0.58  0.572   0.52  3.7 1.9
## X22_MEANS_P                   337 -0.587 -0.58 -0.644  -0.63  4.7 1.9
## X24_NEED_P                    337  0.550  0.55  0.535   0.50  3.6 1.7
## X28_IDENTITY_P                337  0.547  0.56  0.544   0.50  3.8 1.7
## X31_NATURAL_P                 337 -0.321 -0.32 -0.377  -0.38  2.8 1.8
##
## Non missing response frequency for each item
##                                  1    2    3    4    5    6    7 miss
## X9_MANDATORY_P_CRIM           0.19 0.12 0.13 0.16 0.13 0.07 0.20    0
## X10_VOLUNTARY_P_CRIM          0.15 0.09 0.11 0.18 0.14 0.12 0.22    0
## X11_MANDATORY_P_ADULTS        0.29 0.18 0.11 0.14 0.11 0.09 0.09    0
## X12_VOLUNTARY_P_ADULTS        0.07 0.04 0.09 0.14 0.13 0.14 0.39    0
## X13_MANDATORY_P_CHILDREN      0.43 0.14 0.10 0.10 0.08 0.04 0.10    0
## X14_VOLUNTARY_P_CHILDREN      0.28 0.12 0.09 0.16 0.10 0.09 0.16    0
## X19_TRADITIONAL_P             0.03 0.04 0.07 0.16 0.16 0.19 0.35    0
## X20_NATURAL_TRADITIONAL_P     0.18 0.13 0.07 0.16 0.14 0.14 0.18    0
## X25_NATURAL_P                 0.15 0.15 0.15 0.26 0.11 0.08 0.09    0
```

```
## X29_IDENTITY_P                0.12 0.09 0.16 0.28 0.18 0.09 0.09    0
## X30_NATURAL_N                 0.10 0.06 0.09 0.19 0.16 0.18 0.23    0
## X33_MEANS_N                   0.13 0.15 0.13 0.28 0.10 0.10 0.10    0
## X34_GENERAL_P                 0.26 0.13 0.10 0.20 0.16 0.06 0.08    0
## X35_PERSONAL_P                0.49 0.16 0.06 0.11 0.07 0.05 0.06    0
## X36_.BULLY_P                  0.23 0.11 0.07 0.18 0.12 0.11 0.18    0
## X37_BULLY_MANDATORY_P         0.36 0.14 0.09 0.13 0.08 0.07 0.13    0
## X38_PREVENTION_MANDATORY_P    0.36 0.16 0.08 0.14 0.10 0.05 0.10    0
## X39_ALL.CHILDREN_MANDATORY_P  0.61 0.10 0.06 0.11 0.03 0.04 0.04    0
## X40_EVERYBODY_MANDATORY_P     0.51 0.12 0.07 0.13 0.07 0.05 0.05    0
## X41_PERSONAL_P                0.47 0.11 0.07 0.15 0.06 0.05 0.09    0
## X42_PERSONAL_OBJECTIVE_P      0.34 0.10 0.05 0.14 0.13 0.07 0.17    0
## X43_YOUR.CHILD_VOLUNTARY_P    0.34 0.10 0.05 0.14 0.12 0.10 0.15    0
## X44_YOUR.CHILD_MANDATORY_P    0.48 0.10 0.06 0.10 0.07 0.07 0.12    0
## X15_IDENTITY_P                0.16 0.14 0.17 0.22 0.11 0.10 0.09    0
## X17_MEANS_P                   0.09 0.07 0.09 0.28 0.09 0.16 0.23    0
## X18_FREEDOM_P                 0.15 0.14 0.18 0.23 0.10 0.11 0.10    0
## X22_MEANS_P                   0.08 0.09 0.09 0.18 0.17 0.18 0.22    0
## X24_NEED_P                    0.15 0.16 0.18 0.22 0.15 0.08 0.07    0
## X28_IDENTITY_P                0.11 0.12 0.18 0.28 0.12 0.12 0.07    0
## X31_NATURAL_P                 0.32 0.18 0.18 0.16 0.06 0.03 0.07    0
```

```
#Producing the scree plot to help us to determine the number of factors to retain.
scree(survey_data_clean %>% select(mbe_vars), pc = F)
```

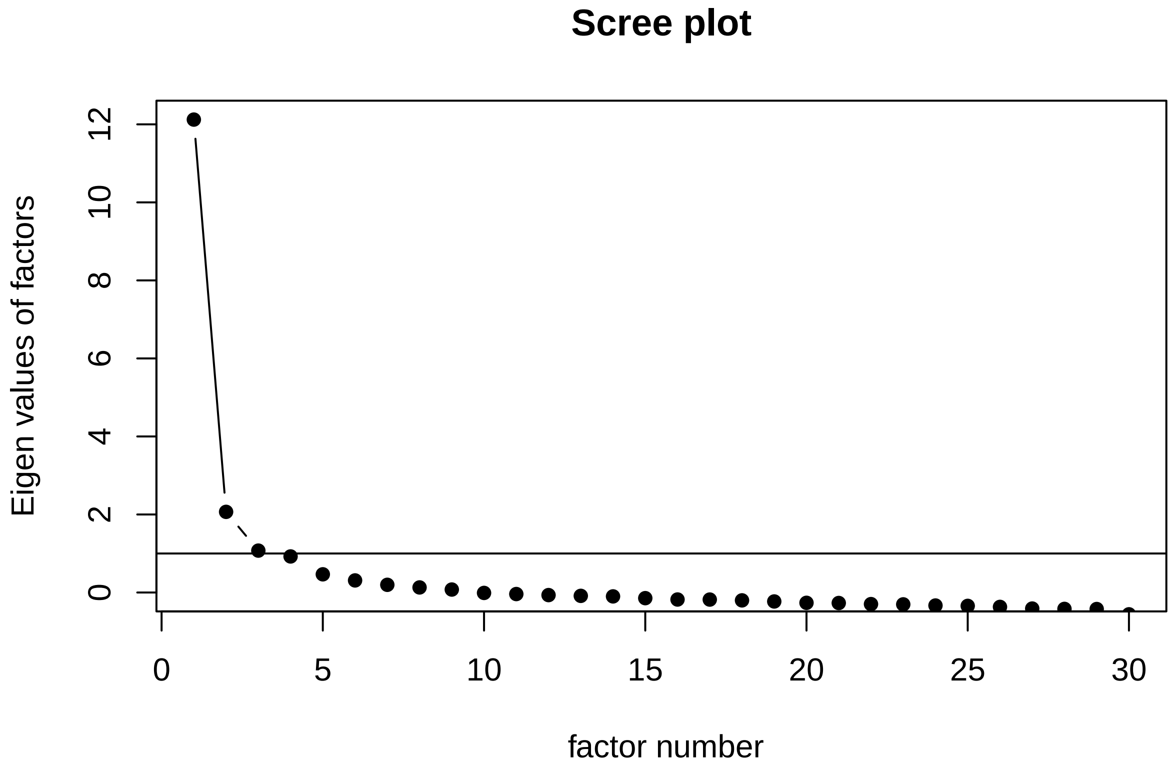


```
#Scree plot for parallel analysis
```

```r
fa.parallel(survey_data_clean %>% select(mbe_vars), fa = "fa")
```

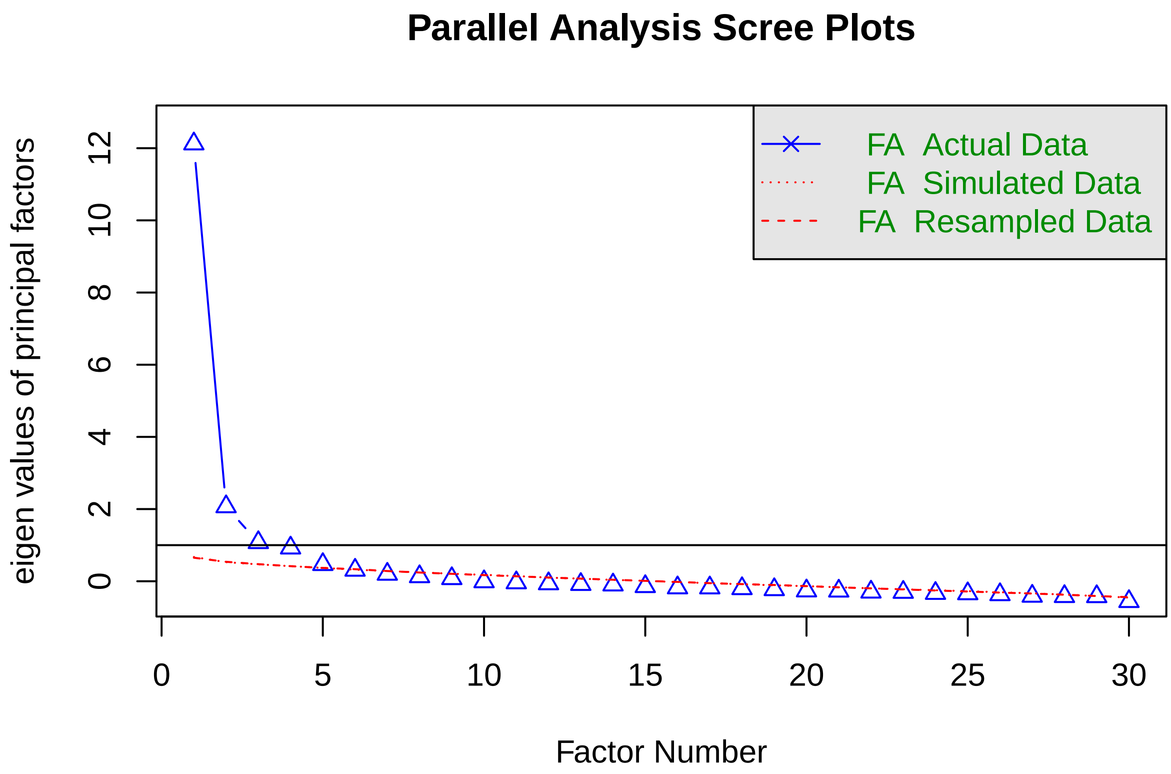


```
## Parallel analysis suggests that the number of factors =  5  and the number of components =  NA
```

```r
#Conducting factor analysis
#fa(survey_data_clean %>% select(mbe_vars), nfactors = 1,rotate = "oblimin", covar = T)
#fa(survey_data_clean %>% select(mbe_vars), nfactors = 2,rotate = "oblimin", covar = T)
#fa(survey_data_clean %>% select(mbe_vars), nfactors = 3,rotate = "oblimin", covar = T)
#fa(survey_data_clean %>% select(mbe_vars), nfactors = 4,rotate = "oblimin", covar = T)
#fa(survey_data_clean %>% select(mbe_vars), nfactors = 5,rotate = "oblimin", covar = T)


#Saving the 4 factor solution
EFA <-
  fa(
    survey_data_clean %>% select(mbe_vars),
    nfactors = 4,
    rotate = "oblimin",
    covar = T
  )

#Calculating comparative fit index (CFI)
fa.CFI(EFA)
```

```
## [1] 0.964347
```

```
#Extracting scores

survey_data_clean$Factor1 <- EFA$scores[, 1]
survey_data_clean$Factor2 <- EFA$scores[, 2]
survey_data_clean$Factor3 <- EFA$scores[, 3]
survey_data_clean$Factor4 <- EFA$scores[, 4]
```

Data normality checks

```
ks_normality_test  <- function(x) {
  ks.test(x,"pnorm", mean=mean(x), sd=sd(x))$p.value
}
sapply(survey_data_clean[,c(5:71, 73:76)], ks_normality_test )
```

```
##         X8_FAMILIARITY_SCALE          X9_MANDATORY_P_CRIM
##                 4.716608e-10                 4.905175e-05
##         X10_VOLUNTARY_P_CRIM       X11_MANDATORY_P_ADULTS
##                 1.721815e-05                 1.404210e-11
##       X12_VOLUNTARY_P_ADULTS     X13_MANDATORY_P_CHILDREN
##                 3.064216e-14                 0.000000e+00
##     X14_VOLUNTARY_P_CHILDREN               X15_IDENTITY_N
##                 9.847191e-09                 1.016638e-04
##                   X16_NEED_P                  X17_MEANS_N
##                 8.889193e-10                 1.317360e-07
##                X18_FREEDOM_N            X19_TRADITIONAL_P
##                 1.787937e-05                 4.086764e-11
##    X20_NATURAL_TRADITIONAL_P                   X21_NEED_N
##                 8.631367e-07                 8.680014e-10
##                  X22_MEANS_N      X23_RISK.OF.COGNITIVE_P
##                 1.944541e-07                 3.765307e-08
##                   X24_NEED_N                X25_NATURAL_P
##                 7.075443e-05                 4.465697e-06
##                X26_FREEDOM_N                X27_FREEDOM_P
##                 1.728575e-05                 4.192329e-06
##               X28_IDENTITY_N               X29_IDENTITY_P
##                 9.318021e-07                 5.475907e-07
##                X30_NATURAL_N                X31_NATURAL_N
##                 8.766466e-08                 1.098618e-09
##      X32_RISK.OF.COGNITIVE_N                  X33_MEANS_N
##                 1.187607e-06                 9.983402e-07
##                X34_GENERAL_P               X35_PERSONAL_P
##                 2.531605e-07                 0.000000e+00
##                 X36_.BULLY_P        X37_BULLY_MANDATORY_P
##                 4.651534e-07                 2.161160e-12
##   X38_PREVENTION_MANDATORY_P X39_ALL.CHILDREN_MANDATORY_P
##                 2.046141e-13                 0.000000e+00
##    X40_EVERYBODY_MANDATORY_P               X41_PERSONAL_P
##                 0.000000e+00                 0.000000e+00
##     X42_PERSONAL_OBJECTIVE_P   X43_YOUR.CHILD_VOLUNTARY_P
##                 1.035949e-12                 8.520962e-13
##   X44_YOUR.CHILD_MANDATORY_P        X45_BULLY_VOLUNTARY_P
##                 0.000000e+00                 9.654377e-11
```

```
##        X46_BULLY_MANDATORY_P   X47_PREVENTION_MANDATORY_P
##                 2.660813e-08                 2.635925e-11
## X48_ALL.CHILDREN_MANDATORY_P    X49_EVERYBODY_MANDATORY_P
##                 0.000000e+00                 0.000000e+00
##                X50_PERSONAL_P     X51_PERSONAL_OBJECTIVE_P
##                 1.223439e-10                 2.856142e-09
##   X52_YOUR.CHILD_VOLUNTARY_P   X53_YOUR.CHILD_MANDATORY_P
##                 3.114790e-07                 2.155054e-12
##            X57_RELIGIOSITY_P               X58_POLITICS_P
##                           NA                 8.744694e-09
##             X59_PATRIOTISM_P           X60_NATIONALISM_P
##                 3.511162e-06                 1.273648e-06
##          X61_INDIVIDUALISM_P       X62_TRADITIONALISM_P
##                 1.126778e-10                 1.971239e-08
##        X63_UTILITARIANISM_P       X64_UTILITARIANISM_P
##                 5.012979e-11                 6.156076e-12
##             X65_DEONTOLOGY_P           X66_DEONTOLOGY_P
##                 1.094986e-04                 3.060199e-09
##             X67_DEONTOLOGY_P                        X2AGE
##                 2.067109e-07                 1.464017e-06
##               X15_IDENTITY_P                  X17_MEANS_P
##                 1.016638e-04                 1.317360e-07
##                X18_FREEDOM_P                  X22_MEANS_P
##                 1.787937e-05                 1.944541e-07
##                   X24_NEED_P                X26_FREEDOM_P
##                 7.075443e-05                 1.728575e-05
##               X28_IDENTITY_P                X31_NATURAL_P
##                 9.318021e-07                 1.098618e-09
##                  X33_MEANS_P                      Factor1
##                 9.983402e-07                 8.052623e-03
##                      Factor2                      Factor3
##                 9.792461e-01                 9.610020e-01
##                      Factor4
##                 7.792211e-01
```

```
gather(survey_data_clean[,c(5:71, 73:76)], factor_key = TRUE) %>%
  ggplot(aes(sample = value)) +
  facet_wrap(~ key , scales = "free") +
  stat_qq() +
  stat_qq_line()
```

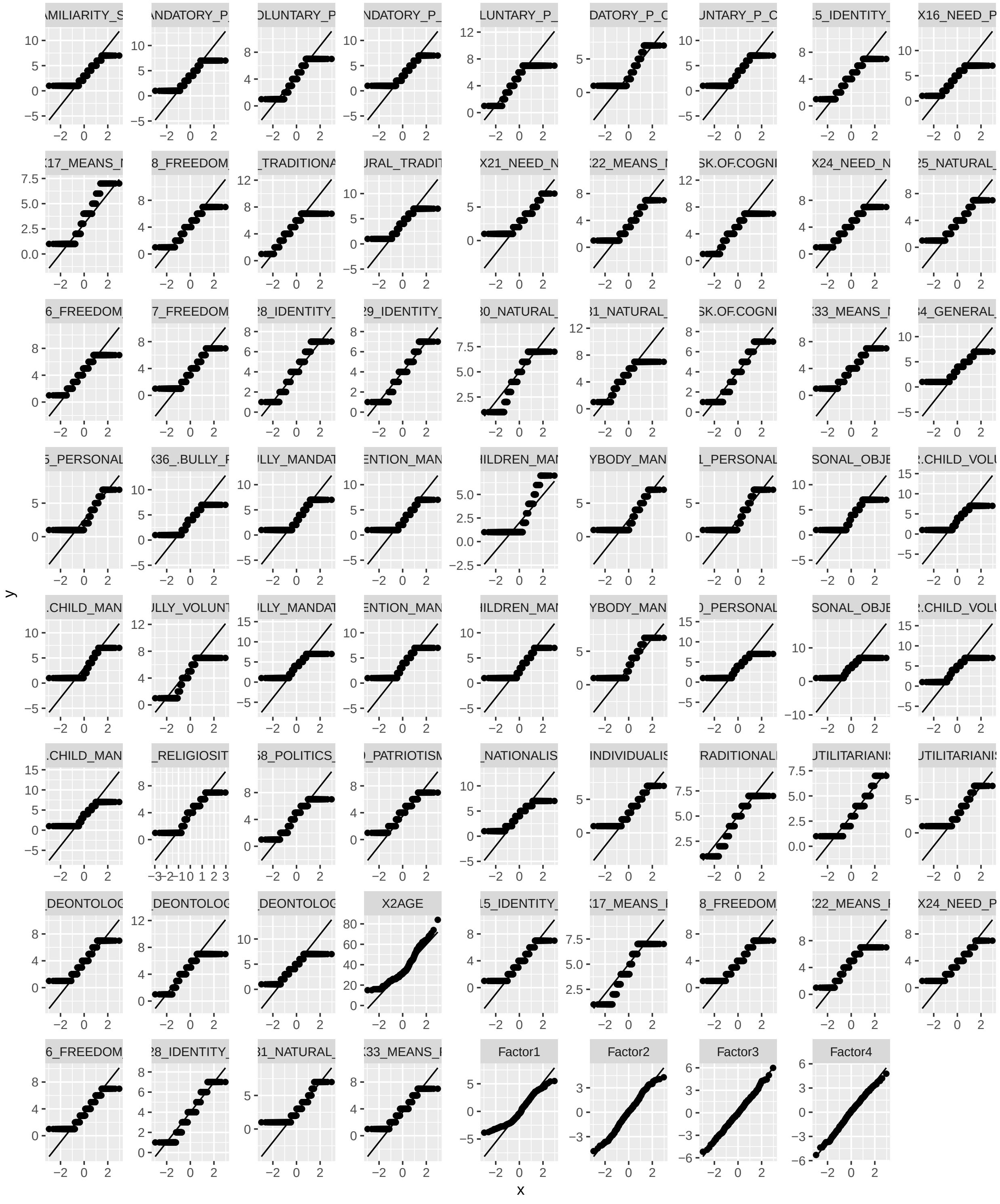

Hypotheses testings

```
#H1 The degree to which members of the public support a ME program depends on
#whether the means employed were pharmacological or non-pharmacological.
```

```
#create composite score for the questions offering pharmacological vs
#nonpharmacological means.

Pharm_list <-
  c(
    "X36_.BULLY_P",
    "X37_BULLY_MANDATORY_P",
    "X38_PREVENTION_MANDATORY_P",
    "X39_ALL.CHILDREN_MANDATORY_P",
    "X40_EVERYBODY_MANDATORY_P",
    "X41_PERSONAL_P",
    "X42_PERSONAL_OBJECTIVE_P"
  )

Other_list <-
  c(
    "X45_BULLY_VOLUNTARY_P",
    "X46_BULLY_MANDATORY_P",
    "X47_PREVENTION_MANDATORY_P",
    "X48_ALL.CHILDREN_MANDATORY_P",
    "X49_EVERYBODY_MANDATORY_P",
    "X50_PERSONAL_P",
    "X51_PERSONAL_OBJECTIVE_P"
  )

Means <- data.frame(
  Pharmacological =  scoreItems(
    survey_data_clean,
    keys = make.keys(survey_data_clean, list(Pharm = Pharm_list)),
    min = 1,
    max = 7
  )$scores[, 1] %>% unname(),
  Other = scoreItems(
    survey_data_clean,
    keys = make.keys(survey_data_clean, list(Other = Other_list)),
    min = 1,
    max = 7
  )$scores[, 1] %>% unname()
) %>% gather(key = "Means")

#Running Wilcoxon two-sample paired signed-rank test

wilcox_test(
  Means,
  formula = value ~ Means,
  paired = T,
  conf.level = 0.95,
  detailed = T
)

## # A tibble: 1 x 12
##   estimate .y.   group1 group2    n1    n2 statistic         p conf.low conf.high
## *    <dbl> <chr> <chr>  <chr>  <int> <int>     <dbl>     <dbl>    <dbl>     <dbl>
```

```
## 1    0.714 value Other  Pharm~   337   337    34206. 6.21e-22    0.571     0.929
## # ... with 2 more variables: method <chr>, alternative <chr>
```

```
wilcox_effsize(
  Means,
  formula = value ~ Means,
  paired = T,
  conf.level = 0.95,
  ci = T
)
```

```
## # A tibble: 1 x 9
##   .y.   group1 group2           effsize    n1    n2 conf.low conf.high magnitude
## * <chr> <chr>  <chr>              <dbl> <int> <int>    <dbl>     <dbl> <ord>
## 1 value Other  Pharmacological   0.519   337   337     0.43       0.6 large
```

```
#Create boxplot visualization for the differation between means (Fig. 2)


means_plot <-
  Means %>% ggplot(aes(x = Means, y = value))+
  scale_y_continuous(limits = c(1, 7), breaks = 1:7)+
  scale_fill_grey()+
  theme_tufte(base_size = 8, base_family = "Helvetica")+
  coord_flip()+
  ylab(NULL)+
  xlab(NULL)+
  stat_summary(
    fun.y = "mean",
    geom = "point",
    colour = "black",
    shape = 15,
    size = 2
  ) + geom_violin() + geom_boxplot(width = 0.1) + stat_summary(
    fun.y = mean,
    geom = "point",
    shape = 18,
    size = 2
  ) + geom_hline(yintercept = 4, linetype = 4)
means_plot
```

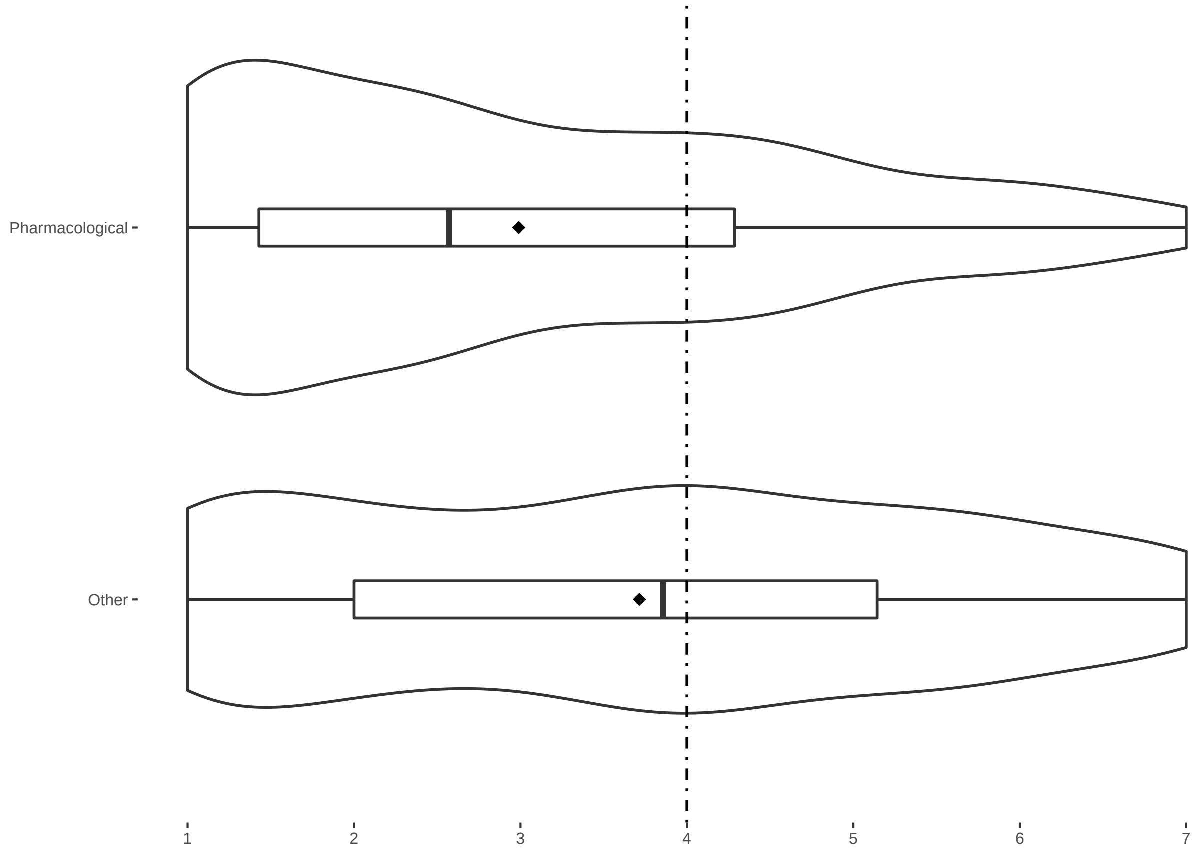


```r
# ggsave(
#   means_plot,
#   file = "Fig2.eps",
#   device = "eps",
#   width = 84,
#   height = 56,
#   units = "mm"
# )
# ggsave(
#   means_plot,
#   file = "Fig2.png",
#   width = 84,
#   height = 56,
#   units = "mm"
# )

#Only the variables ask for support are included in form the composite score

total_score_keys <-
  make.keys(survey_data_clean, list(TotalScore = which(
    colnames(survey_data_clean) %in% c(
      "X9_MANDATORY_P_CRIM",
      "X10_VOLUNTARY_P_CRIM",
      "X11_MANDATORY_P_ADULTS",
      "X12_VOLUNTARY_P_ADULTS",
      "X13_MANDATORY_P_CHILDREN",
```

```
      "X14_VOLUNTARY_P_CHILDREN",
      "X15_IDENTITY_P",
      "X17_MEANS_P",
      "X18_FREEDOM_P",
      "X19_TRADITIONAL_P",
      "X20_NATURAL_TRADITIONAL_P",
      "X22_MEANS_P",
      "X24_NEED_P",
      "X25_NATURAL_P",
      "X27_FREEDOM_P",
      "X28_IDENTITY_P",
      "X29_IDENTITY_P",
      "X30_NATURAL_N",
      "X31_NATURAL_N",
      "X33_MEANS_P",
      "X36_.BULLY_P",
      "X37_BULLY_MANDATORY_P",
      "X38_PREVENTION_MANDATORY_P",
      "X39_ALL.CHILDREN_MANDATORY_P",
      "X40_EVERYBODY_MANDATORY_P",
      "X41_PERSONAL_P",
      "X42_PERSONAL_OBJECTIVE_P",
      "X43_YOUR.CHILD_VOLUNTARY_P",
      "X44_YOUR.CHILD_MANDATORY_P"
    )
  )))

totalscores <-
  scoreItems(survey_data_clean,
             keys = total_score_keys,
             min = 1,
             max = 7)

survey_data_clean$TotalScore <- totalscores$scores

#H2 The level of support for MBE will differ between people familiar with the term MBE.
#Running Wilcoxon-Mann-Whitney test

wilcox_test(
  data = survey_data_clean,
  formula = TotalScore ~ X7_FAMILIARITY,
  detailed = T,
  exact = T,
  conf.level = 0.95
)

## # A tibble: 1 x 12
##     estimate .y.         group1 group2    n1    n2 statistic     p conf.low conf.high
## *      <dbl> <chr>       <chr>  <chr>  <int> <int>     <dbl> <dbl>    <dbl>     <dbl>
## 1 -0.0000388 TotalScore 1      2         92   245    11182. 0.912   -0.241     0.207
## # ... with 2 more variables: method <chr>, alternative <chr>

wilcox_effsize(
  data = survey_data_clean,
```

```
  formula = TotalScore ~ X7_FAMILIARITY,
  conf.level = 0.95,
  ci = T
)

## # A tibble: 1 x 9
##   .y.         group1 group2 effsize    n1    n2 conf.low conf.high magnitude
## * <chr>       <chr>  <chr>    <dbl> <int> <int>    <dbl>     <dbl> <ord>
## 1 TotalScore 1      2      0.00605    92   245   0.0014      0.13 small

survey_data_clean$FAMILIARITY <-
  survey_data_clean$X7_FAMILIARITY %>% as.factor
levels(survey_data_clean$FAMILIARITY) <- c("Familiar", "Unfamiliar")

#Create boxplot visualization for showing difference between groups (Fig. 3)

familiriaty_plot <-
  survey_data_clean %>% ggplot(aes(x = as.factor(FAMILIARITY), y = TotalScore)) +
  scale_y_continuous(limits = c(1, 7), breaks = 1:7)+
  scale_fill_grey()+
  theme_tufte(base_size = 8, base_family = "Helvetica")+
  coord_flip()+
  ylab(NULL)+
  xlab(NULL)+
  stat_summary(
    fun.y = "mean",
    geom = "point",
    colour = "black",
    shape = 15,
    size = 2
  ) + geom_violin() + geom_boxplot(width = 0.1) + stat_summary(
    fun.y = mean,
    geom = "point",
    shape = 18,
    size = 2
  ) + geom_hline(yintercept = 4, linetype = 4)
familiriaty_plot
```

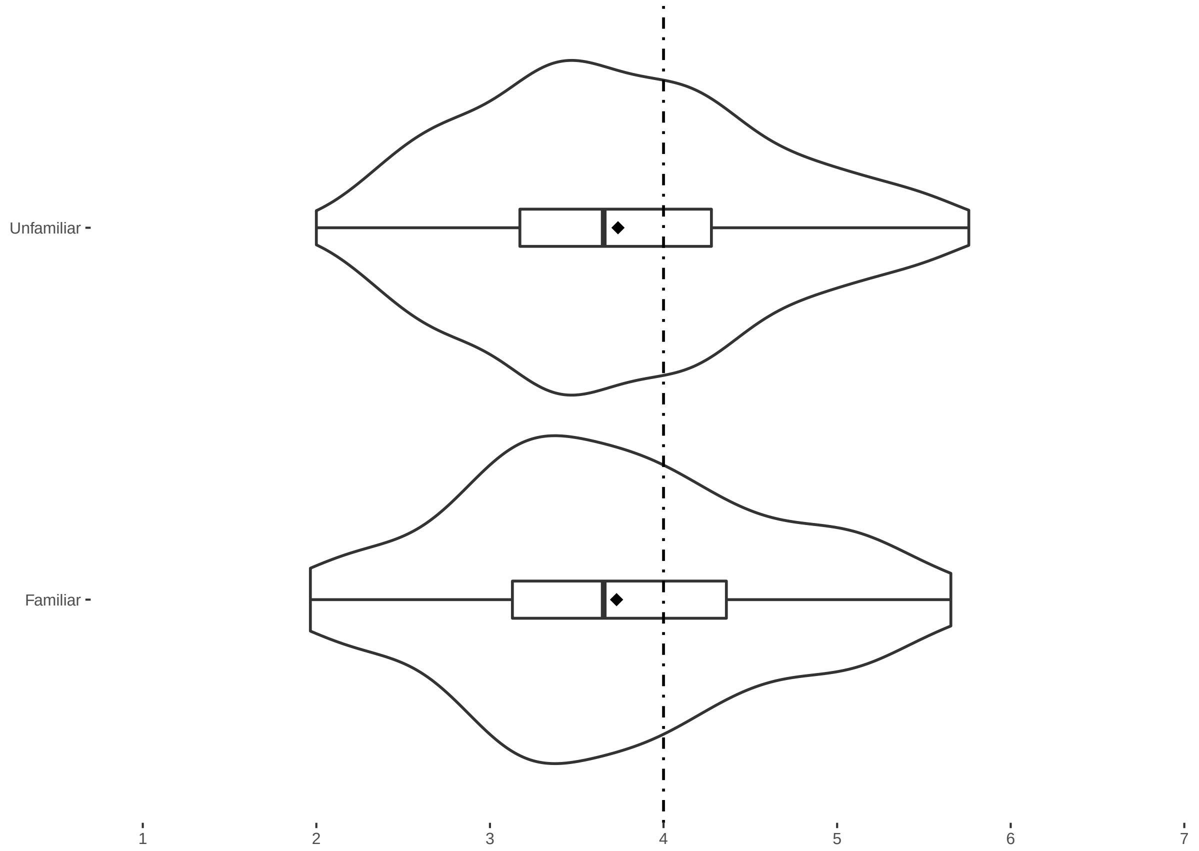


```
# ggsave(
#   familiriaty_plot,
#   file = "Fig3.eps",
#   device = "eps",
#   width = 84,
#   height = 56,
#   units = "mm"
# )
# ggsave(
#   familiriaty_plot,
#   file = "Fig3.png",
#   width = 84,
#   height = 56,
#   units = "mm"
# )

#H3 People's attitudes towards MBE differ among people with different levels of education.

survey_data_clean$Univeristy_degree <- "No Degree"
survey_data_clean$Univeristy_degree[survey_data_clean$X6_EDU == 7] <-
  "Bachelor's Degree"
survey_data_clean$Univeristy_degree[survey_data_clean$X6_EDU > 7] <-
  "Advanced Degree"

survey_data_clean %>% group_by(Univeristy_degree) %>% shapiro_test(TotalScore)
```

```
## # A tibble: 3 x 4
##   Univeristy_degree variable    statistic      p
##   <chr>             <chr>           <dbl>  <dbl>
## 1 Advanced Degree   TotalScore      0.979 0.0498
## 2 Bachelor's Degree TotalScore      0.978 0.0871
## 3 No Degree         TotalScore      0.973 0.0247
```

```
kruskal_test(data = survey_data_clean, formula = TotalScore ~ Univeristy_degree)
```

```
## # A tibble: 1 x 6
##   .y.             n statistic    df       p method
## * <chr>       <int>     <dbl> <int>   <dbl> <chr>
## 1 TotalScore    337      10.9     2 0.00434 Kruskal-Wallis
```

```
kruskal_effsize(
  data = survey_data_clean,
  formula = TotalScore ~ Univeristy_degree,
  conf.level = 0.95,
  ci = T
)
```

```
## # A tibble: 1 x 7
##   .y.             n effsize conf.low conf.high method  magnitude
## * <chr>       <int>   <dbl>    <dbl>     <dbl> <chr>   <ord>
## 1 TotalScore    337  0.0266   0.0006      0.08 eta2[H] small
```

```
dunn_test(data = survey_data_clean, formula = TotalScore ~ Univeristy_degree)
```

```
## # A tibble: 3 x 9
##   .y.        group1   group2     n1    n2 statistic       p   p.adj p.adj.signif
## * <chr>      <chr>    <chr>   <int> <int>     <dbl>   <dbl>   <dbl> <chr>
## 1 TotalScore Advance~ Bachel~   126   101     2.45  0.0145  0.0290  *
## 2 TotalScore Advance~ No Deg~   126   110     3.08  0.00204 0.00611 **
## 3 TotalScore Bachelo~ No Deg~   101   110     0.551 0.582   0.582   ns
```

```
#Create boxplot visualization for showing difference between groups (Fig. 4)

education_plot <-
  survey_data_clean %>% ggplot(aes(x = Univeristy_degree, y = TotalScore)) +
  scale_y_continuous(limits = c(1, 7), breaks = 1:7)  + scale_fill_grey() +
  theme_tufte(base_size = 8, base_family = "Helvetica") + coord_flip() + ylab(NULL) +
  xlab(NULL) +
  stat_summary(
    fun.y = "mean",
    geom = "point",
    colour = "black",
    shape = 15,
    size = 2
  ) + geom_violin() + geom_boxplot(width = 0.1) + stat_summary(
    fun.y = mean,
    geom = "point",
    shape = 18,
```

```
    size = 2
  ) + geom_hline(yintercept = 4, linetype = 4)
education_plot
```

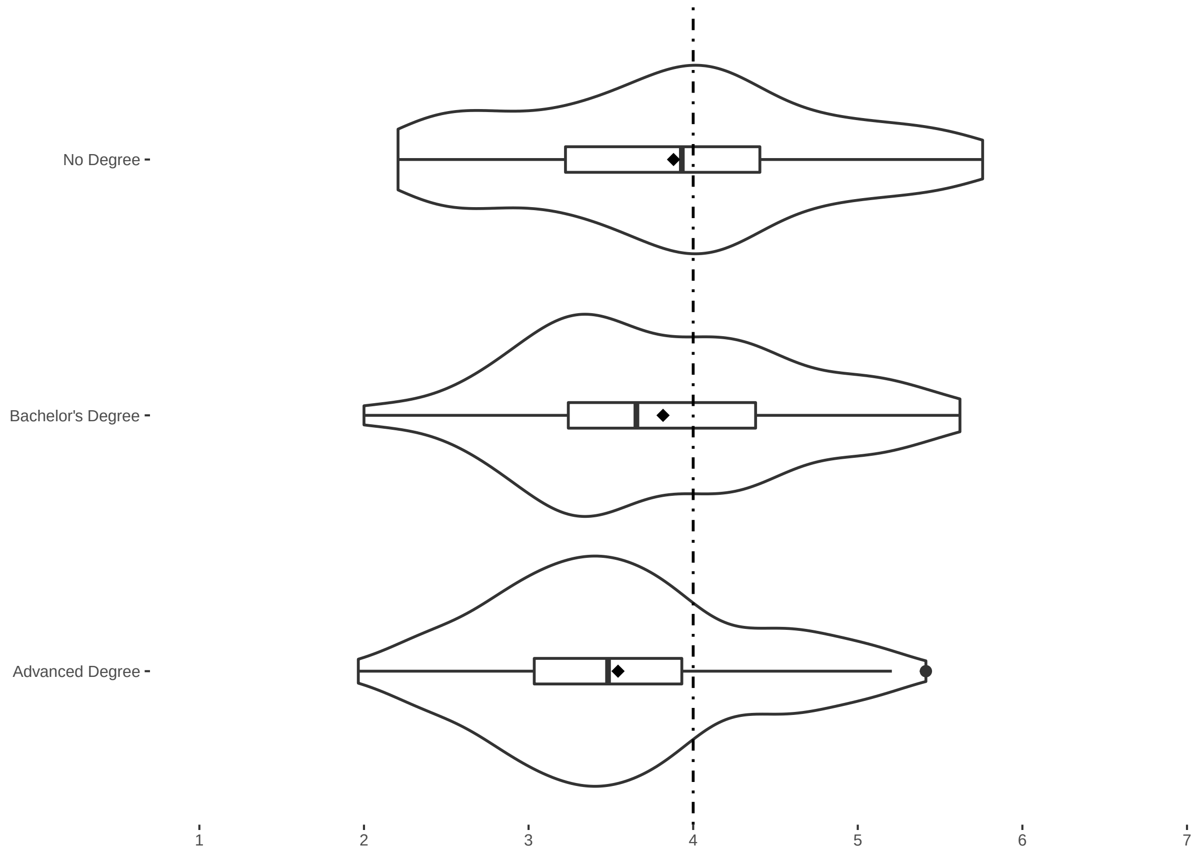


```
# ggsave(
#   education_plot,
#   file = "Fig4.eps",
#   device = "eps",
#   width = 84,
#   height = 84,
#   units = "mm"
# )
# ggsave(
#   education_plot,
#   file = "Fig4.png",
#   width = 84,
#   height = 84,
#   units = "mm"
# )

#H4 People's attitudes towards MBE differ among people based on whether they are
#more deontological or utilitarian
survey_data_clean$X63_UTILITARIANISM_N<- reverse_likert(survey_data_clean$X63_UTILITARIANISM_P)
survey_data_clean$X64_UTILITARIANISM_N <- reverse_likert(survey_data_clean$X64_UTILITARIANISM_P)
```

```
Deont_Util_list <-
  c(
    "X65_DEONTOLOGY_P",
    "X66_DEONTOLOGY_P",
    "X67_DEONTOLOGY_P",
    "X63_UTILITARIANISM_N",
    "X64_UTILITARIANISM_N"
  )

survey_data_clean$Deontology_Utilitarianism <-
  scoreItems(
    survey_data_clean,
    keys = make.keys(survey_data_clean, list(Deont_Util = Deont_Util_list)),
    min = 1,
    max = 7,
  )$scores[, 1] %>% unname()



median_DeontUtil <-
  median(survey_data_clean$X63_UTILITARIANISM_P)
survey_data_clean$Deont_Util_class[survey_data_clean$X63_UTILITARIANISM_P <
                                     median_DeontUtil] <- "Deontologically\nOriented"
survey_data_clean$Deont_Util_class[survey_data_clean$X63_UTILITARIANISM_P >=
                                     median_DeontUtil] <- "Prone to Utilitarian\nThinking"

wilcox_test(
  data = survey_data_clean,
  formula = TotalScore ~ Deont_Util_class,
  detailed = T,
  exact = T,
  conf.level = 0.95
)

## # A tibble: 1 x 12
##   estimate .y.        group1 group2    n1    n2 statistic       p conf.low conf.high
## *    <dbl> <chr>      <chr>  <chr>  <int> <int>     <dbl>   <dbl>    <dbl>     <dbl>
## 1   -0.414 TotalScore "Deon~ "Pron~   163   174     10177 7.47e-6   -0.621    -0.241
## # ... with 2 more variables: method <chr>, alternative <chr>

cor.test(survey_data_clean$X63_UTILITARIANISM_P,survey_data_clean$TotalScore, conf.level = 0.95, method

##
##  Kendall's rank correlation tau
##
## data:  survey_data_clean$X63_UTILITARIANISM_P and survey_data_clean$TotalScore
## z = 4.9446, p-value = 7.63e-07
## alternative hypothesis: true tau is not equal to 0
## sample estimates:
##       tau
## 0.1983358

cor_test(survey_data_clean, vars=c(TotalScore, X63_UTILITARIANISM_P), method = "kendall", conf.level = 
```

```
## # A tibble: 1 x 6
##   var1       var2                  cor statistic           p method
##   <chr>      <chr>               <dbl>     <dbl>       <dbl> <chr>
## 1 TotalScore X63_UTILITARIANISM_P   0.2      4.94 0.000000763 Kendall
```

```
util_plot <-
  survey_data_clean %>% ggplot(aes(x = Deont_Util_class, y = TotalScore))+
  scale_y_continuous(limits = c(1, 7), breaks = 1:7)+
  scale_fill_grey()+
  theme_tufte(base_size = 8, base_family = "Helvetica")+
  coord_flip()+
  ylab(NULL)+
  xlab(NULL)+
  stat_summary(
    fun.y = "mean",
    geom = "point",
    colour = "black",
    shape = 15,
    size = 2
  )+
  geom_violin()+
  geom_boxplot(width = 0.1)+
  stat_summary(
    fun.y = mean,
    geom = "point",
    shape = 18,
    size = 2
  )+
  geom_hline(yintercept = 4, linetype = 4)
util_plot
```

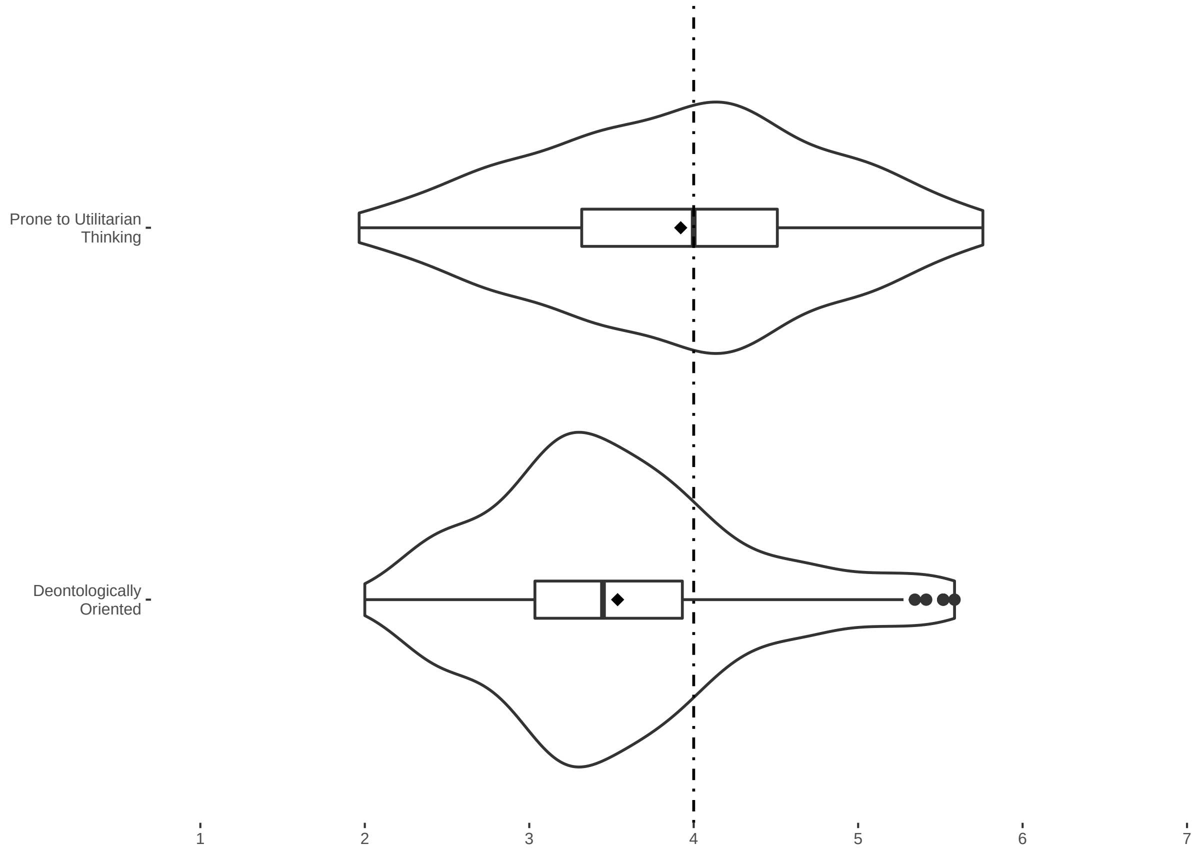


```
# ggsave(
#   util_plot,
#   file = "Fig5.eps",
#   device = "eps",
#   width = 84,
#   height = 56,
#   units = "mm"
# )
# ggsave(
#   util_plot,
#   file = "Fig5.png",
#   width = 84,
#   height = 56,
#   units = "mm"
# )


#ODDS RATIOS given in Table 3

#Designate higher than median observations for each factor HTMF

survey_data_clean$HTMFactor1 <-
  ifelse(survey_data_clean$Factor1 > median(survey_data_clean$Factor1),
         1,
         0)
survey_data_clean$HTMFactor2 <-
```

```
  ifelse(survey_data_clean$Factor2 > median(survey_data_clean$Factor2),
         1,
         0)
survey_data_clean$HTMFactor3 <-
  ifelse(survey_data_clean$Factor3 > median(survey_data_clean$Factor3),
         1,
         0)
survey_data_clean$HTMFactor4 <-
  ifelse(survey_data_clean$Factor4 > median(survey_data_clean$Factor4),
         1,
         0)


#Age

#Younger than 25 vs 25-30
survey_data_clean$AGE <- NA
survey_data_clean$AGE[survey_data_clean$X2AGE < 25] <- 1
survey_data_clean$AGE[survey_data_clean$X2AGE >= 25 &
                        survey_data_clean$X2AGE < 30] <- 2
epitools::oddsratio(survey_data_clean$HTMFactor1,
                    y = survey_data_clean$AGE ,
                    correction = T)

## $data
##          Outcome
## Predictor  1  2 Total
##     0     23 43    66
##     1     35 35    70
##     Total 58 78   136
##
## $measure
##          odds ratio with 95% C.I.
## Predictor  estimate     lower    upper
##         0 1.0000000        NA       NA
##         1 0.5383052 0.2665882 1.071547
##
## $p.value
##          two-sided
## Predictor midp.exact fisher.exact chi.square
##         0         NA           NA         NA
##         1 0.07803277   0.08472277  0.1069303
##
## $correction
## [1] TRUE
##
## attr(,"method")
## [1] "median-unbiased estimate & mid-p exact CI"

epitools::oddsratio(survey_data_clean$HTMFactor2,
                    y = survey_data_clean$AGE,
                    correction = T)

## $data
```

```
##          Outcome
## Predictor  1  2 Total
##     0     24 42    66
##     1     34 36    70
##     Total 58 78   136
##
## $measure
##          odds ratio with 95% C.I.
## Predictor  estimate     lower    upper
##         0 1.0000000        NA       NA
##         1 0.6081585 0.3026311 1.208076
##
## $p.value
##          two-sided
## Predictor midp.exact fisher.exact chi.square
##         0         NA           NA         NA
##         1  0.1560228    0.1681741  0.2057896
##
## $correction
## [1] TRUE
##
## attr(,"method")
## [1] "median-unbiased estimate & mid-p exact CI"
```

```
epitools::oddsratio(survey_data_clean$HTMFactor3,
                    y = survey_data_clean$AGE,
                    correction = T)
```

```
## $data
##          Outcome
## Predictor  1  2 Total
##     0     25 52    77
##     1     33 26    59
##     Total 58 78   136
##
## $measure
##          odds ratio with 95% C.I.
## Predictor  estimate     lower     upper
##         0 1.0000000        NA        NA
##         1 0.3825028 0.1868611 0.7685839
##
## $p.value
##          two-sided
## Predictor  midp.exact fisher.exact chi.square
##         0          NA           NA         NA
##         1 0.006799771  0.008538658 0.01025101
##
## $correction
## [1] TRUE
##
## attr(,"method")
## [1] "median-unbiased estimate & mid-p exact CI"
```

```
epitools::oddsratio(survey_data_clean$HTMFactor4,
```

```
                    y = survey_data_clean$AGE,
                    correction = T)

## $data
##          Outcome
## Predictor  1  2 Total
##     0     31 35    66
##     1     27 43    70
##     Total 58 78   136
##
## $measure
##          odds ratio with 95% C.I.
## Predictor estimate     lower    upper
##         0  1.00000        NA       NA
##         1  1.40587 0.7091281 2.805522
##
## $p.value
##          two-sided
## Predictor midp.exact fisher.exact chi.square
##         0         NA           NA         NA
##         1  0.3295525      0.38648  0.4143422
##
## $correction
## [1] TRUE
##
## attr(,"method")
## [1] "median-unbiased estimate & mid-p exact CI"

#Younger than 25 vs 30-35
survey_data_clean$AGE <- NA
survey_data_clean$AGE[survey_data_clean$X2AGE < 25] <- 1
survey_data_clean$AGE[survey_data_clean$X2AGE >= 30 &
                        survey_data_clean$X2AGE < 35] <- 2
epitools::oddsratio(survey_data_clean$HTMFactor1,
                    y = survey_data_clean$AGE ,
                    correction = T)

## $data
##          Outcome
## Predictor  1  2 Total
##     0     23 36    59
##     1     35 34    69
##     Total 58 70   128
##
## $measure
##          odds ratio with 95% C.I.
## Predictor  estimate     lower   upper
##         0 1.0000000        NA      NA
##         1 0.6238733 0.3046864 1.26227
##
## $p.value
##          two-sided
## Predictor midp.exact fisher.exact chi.square
```

```
##         0         NA           NA         NA
##         1  0.1900685    0.2144156  0.2492783
##
## $correction
## [1] TRUE
##
## attr(,"method")
## [1] "median-unbiased estimate & mid-p exact CI"
```

```
epitools::oddsratio(survey_data_clean$HTMFactor2,
                    y = survey_data_clean$AGE,
                    correction = T)
```

```
## $data
##          Outcome
## Predictor  1  2 Total
##     0     24 36    60
##     1     34 34    68
##     Total 58 70   128
##
## $measure
##          odds ratio with 95% C.I.
## Predictor  estimate    lower    upper
##         0 1.0000000       NA       NA
##         1 0.6696012 0.328246 1.352436
##
## $p.value
##          two-sided
## Predictor midp.exact fisher.exact chi.square
##         0         NA           NA         NA
##         1  0.2641121    0.2887512  0.3389472
##
## $correction
## [1] TRUE
##
## attr(,"method")
## [1] "median-unbiased estimate & mid-p exact CI"
```

```
epitools::oddsratio(survey_data_clean$HTMFactor3,
                    y = survey_data_clean$AGE,
                    correction = T)
```

```
## $data
##          Outcome
## Predictor  1  2 Total
##     0     25 37    62
##     1     33 33    66
##     Total 58 70   128
##
## $measure
##          odds ratio with 95% C.I.
## Predictor  estimate     lower    upper
##         0 1.0000000        NA       NA
```

```
##         1 0.6785059 0.3333788 1.368575
##
## $p.value
##          two-sided
## Predictor midp.exact fisher.exact chi.square
##         0         NA           NA         NA
##         1  0.2791512    0.2914768  0.3567707
##
## $correction
## [1] TRUE
##
## attr(,"method")
## [1] "median-unbiased estimate & mid-p exact CI"
```

```
epitools::oddsratio(survey_data_clean$HTMFactor4,
                   y = survey_data_clean$AGE,
                   correction = T)
```

```
## $data
##          Outcome
## Predictor  1  2 Total
##     0     31 35    66
##     1     27 35    62
##     Total 58 70   128
##
## $measure
##          odds ratio with 95% C.I.
## Predictor estimate    lower    upper
##         0 1.000000       NA       NA
##         1 1.146427 0.568709 2.319015
##
## $p.value
##          two-sided
## Predictor midp.exact fisher.exact chi.square
##         0         NA           NA         NA
##         1  0.7023342    0.7253017  0.8329232
##
## $correction
## [1] TRUE
##
## attr(,"method")
## [1] "median-unbiased estimate & mid-p exact CI"
```

```
#Younger than 25 vs 35 and older
survey_data_clean$AGE <- NA
survey_data_clean$AGE[survey_data_clean$X2AGE < 25] <- 1
survey_data_clean$AGE[survey_data_clean$X2AGE >= 35] <- 2
epitools::oddsratio(survey_data_clean$HTMFactor1,
                   y = survey_data_clean$AGE ,
                   correction = T)
```

```
## $data
##          Outcome
```

```
## Predictor  1   2 Total
##     0     23  67    90
##     1     35  64    99
##     Total 58 131   189
##
## $measure
##          odds ratio with 95% C.I.
## Predictor  estimate     lower    upper
##         0 1.0000000        NA       NA
##         1 0.6303601 0.3323844 1.178679
##
## $p.value
##          two-sided
## Predictor midp.exact fisher.exact chi.square
##         0         NA           NA         NA
##         1  0.1490477    0.1580279  0.1933367
##
## $correction
## [1] TRUE
##
## attr(,"method")
## [1] "median-unbiased estimate & mid-p exact CI"
```

```
epitools::oddsratio(survey_data_clean$HTMFactor2,
                    y = survey_data_clean$AGE,
                    correction = T)
```

```
## $data
##          Outcome
## Predictor  1   2 Total
##     0     24  67    91
##     1     34  64    98
##     Total 58 131   189
##
## $measure
##          odds ratio with 95% C.I.
## Predictor  estimate     lower    upper
##         0 1.0000000        NA       NA
##         1 0.6766712 0.3583382 1.262896
##
## $p.value
##          two-sided
## Predictor midp.exact fisher.exact chi.square
##         0         NA           NA         NA
##         1  0.2206527    0.2693336  0.2795182
##
## $correction
## [1] TRUE
##
## attr(,"method")
## [1] "median-unbiased estimate & mid-p exact CI"
```

```
epitools::oddsratio(survey_data_clean$HTMFactor3,
```

```
                    y = survey_data_clean$AGE,
                    correction = T)
```

```
## $data
##          Outcome
## Predictor  1   2 Total
##     0     25  55    80
##     1     33  76   109
##     Total 58 131   189
##
## $measure
##          odds ratio with 95% C.I.
## Predictor estimate     lower   upper
##         0 1.000000        NA      NA
##         1 1.047459 0.5560007 1.95966
##
## $p.value
##          two-sided
## Predictor midp.exact fisher.exact chi.square
##         0         NA           NA         NA
##         1   0.884989            1          1
##
## $correction
## [1] TRUE
##
## attr(,"method")
## [1] "median-unbiased estimate & mid-p exact CI"
```

```
epitools::oddsratio(survey_data_clean$HTMFactor4,
                    y = survey_data_clean$AGE,
                    correction = T)
```

```
## $data
##          Outcome
## Predictor  1   2 Total
##     0     31  68    99
##     1     27  63    90
##     Total 58 131   189
##
## $measure
##          odds ratio with 95% C.I.
## Predictor estimate     lower    upper
##         0 1.000000        NA       NA
##         1 1.062879 0.5704678 1.988076
##
## $p.value
##          two-sided
## Predictor midp.exact fisher.exact chi.square
##         0         NA           NA         NA
##         1  0.8477183    0.8755672   0.970011
##
## $correction
## [1] TRUE
```

```
##
## attr(,"method")
## [1] "median-unbiased estimate & mid-p exact CI"
```

```
#Sex

epitools::oddsratio(
  survey_data_clean$HTMFactor1,
  y = survey_data_clean$X1..Pol,
  rev = "columns",
  correction = T
)
```

```
## $data
##          Outcome
## Predictor   2   1 Total
##     0     117  52   169
##     1     118  50   168
##     Total 235 102   337
##
## $measure
##          odds ratio with 95% C.I.
## Predictor  estimate     lower   upper
##         0 1.0000000        NA      NA
##         1 0.9536101 0.5976545 1.52051
##
## $p.value
##          two-sided
## Predictor midp.exact fisher.exact chi.square
##         0         NA           NA         NA
##         1  0.8416973    0.9057158  0.9341029
##
## $correction
## [1] TRUE
##
## attr(,"method")
## [1] "median-unbiased estimate & mid-p exact CI"
```

```
epitools::oddsratio(
  survey_data_clean$HTMFactor2,
  y = survey_data_clean$X1..Pol,
  rev = "columns",
  correction = T
)
```

```
## $data
##          Outcome
## Predictor   2   1 Total
##     0     120  49   169
##     1     115  53   168
##     Total 235 102   337
##
## $measure
```

```
##          odds ratio with 95% C.I.
## Predictor estimate     lower    upper
##         0 1.000000        NA       NA
##         1 1.128038 0.7075988 1.801053
##
## $p.value
##          two-sided
## Predictor midp.exact fisher.exact chi.square
##         0         NA           NA         NA
##         1  0.6125176    0.6365571  0.6953502
##
## $correction
## [1] TRUE
##
## attr(,"method")
## [1] "median-unbiased estimate & mid-p exact CI"
```

```
epitools::oddsratio(
  survey_data_clean$HTMFactor3,
  y = survey_data_clean$X1..Pol,
  rev = "columns" ,
  correction = T
)
```

```
## $data
##          Outcome
## Predictor   2   1 Total
##     0     108  61   169
##     1     127  41   168
##     Total 235 102   337
##
## $measure
##          odds ratio with 95% C.I.
## Predictor  estimate     lower     upper
##         0 1.0000000        NA        NA
##         1 0.5731011 0.3553811 0.9168751
##
## $p.value
##          two-sided
## Predictor midp.exact fisher.exact chi.square
##         0         NA           NA         NA
##         1 0.02013039   0.02410901 0.02662423
##
## $correction
## [1] TRUE
##
## attr(,"method")
## [1] "median-unbiased estimate & mid-p exact CI"
```

```
epitools::oddsratio(
  survey_data_clean$HTMFactor4,
  y = survey_data_clean$X1..Pol,
  rev = "columns" ,
```

```
  correction = T
)

## $data
##          Outcome
## Predictor   2   1 Total
##     0     121  48   169
##     1     114  54   168
##     Total 235 102   337
##
## $measure
##          odds ratio with 95% C.I.
## Predictor estimate     lower    upper
##         0 1.000000        NA       NA
##         1 1.193106 0.7484142 1.906358
##
## $p.value
##          two-sided
## Predictor midp.exact fisher.exact chi.square
##         0         NA           NA         NA
##         1  0.4581019    0.4784107  0.5295132
##
## $correction
## [1] TRUE
##
## attr(,"method")
## [1] "median-unbiased estimate & mid-p exact CI"

#Education Univeristy degree

#No degree vs Bachelor degree
survey_data_clean$Univeristy_degree <- NA
survey_data_clean$Univeristy_degree[survey_data_clean$X6_EDU < 7] <-
  1
survey_data_clean$Univeristy_degree[survey_data_clean$X6_EDU == 7] <-
  2
epitools::oddsratio(
  survey_data_clean$HTMFactor1,
  y = survey_data_clean$Univeristy_degree ,
  correction = T
)

## $data
##          Outcome
## Predictor   1   2 Total
##     0      42  53    95
##     1      68  48   116
##     Total 110 101   211
##
## $measure
##          odds ratio with 95% C.I.
## Predictor  estimate     lower     upper
##         0 1.0000000        NA        NA
```

```
##         1 0.5614447 0.3224541 0.9706121
##
## $p.value
##          two-sided
## Predictor midp.exact fisher.exact chi.square
##         0         NA           NA         NA
##         1 0.03872052    0.0391605 0.05163038
##
## $correction
## [1] TRUE
##
## attr(,"method")
## [1] "median-unbiased estimate & mid-p exact CI"
```

```
epitools::oddsratio(
  survey_data_clean$HTMFactor2,
  y = survey_data_clean$Univeristy_degree ,
  correction = T
)
```

```
## $data
##          Outcome
## Predictor   1   2 Total
##     0      53  58   111
##     1      57  43   100
##     Total 110 101   211
##
## $measure
##          odds ratio with 95% C.I.
## Predictor estimate     lower    upper
##         0 1.000000        NA       NA
##         1 0.690981 0.3991365 1.190336
##
## $p.value
##          two-sided
## Predictor midp.exact fisher.exact chi.square
##         0         NA           NA         NA
##         1  0.1831298    0.2144567   0.228063
##
## $correction
## [1] TRUE
##
## attr(,"method")
## [1] "median-unbiased estimate & mid-p exact CI"
```

```
epitools::oddsratio(
  survey_data_clean$HTMFactor3,
  y = survey_data_clean$Univeristy_degree ,
  correction = T
)
```

```
## $data
##          Outcome
```

```
## Predictor   1   2 Total
##     0      48  47    95
##     1      62  54   116
##     Total 110 101   211
##
## $measure
##          odds ratio with 95% C.I.
## Predictor  estimate     lower    upper
##         0 1.0000000        NA       NA
##         1 0.8901144 0.5154221 1.535466
##
## $p.value
##          two-sided
## Predictor midp.exact fisher.exact chi.square
##         0         NA           NA         NA
##         1  0.6754311    0.6804669  0.7762435
##
## $correction
## [1] TRUE
##
## attr(,"method")
## [1] "median-unbiased estimate & mid-p exact CI"
```

```
epitools::oddsratio(
  survey_data_clean$HTMFactor4,
  y = survey_data_clean$Univeristy_degree ,
  correction = T
)
```

```
## $data
##          Outcome
## Predictor   1   2 Total
##     0      57  43   100
##     1      53  58   111
##     Total 110 101   211
##
## $measure
##          odds ratio with 95% C.I.
## Predictor estimate     lower    upper
##         0 1.000000        NA       NA
##         1 1.447175 0.8400987 2.505409
##
## $p.value
##          two-sided
## Predictor midp.exact fisher.exact chi.square
##         0         NA           NA         NA
##         1  0.1831298    0.2144567   0.228063
##
## $correction
## [1] TRUE
##
## attr(,"method")
## [1] "median-unbiased estimate & mid-p exact CI"
```

```
#No degree vs Advanced degree
survey_data_clean$Univeristy_degree <- NA
survey_data_clean$Univeristy_degree[survey_data_clean$X6_EDU < 7] <-
  1
survey_data_clean$Univeristy_degree[survey_data_clean$X6_EDU > 7] <-
  2
epitools::oddsratio(
  survey_data_clean$HTMFactor1,
  y = survey_data_clean$Univeristy_degree ,
  correction = T
)
```

```
## $data
##          Outcome
## Predictor   1   2 Total
##     0      42  74   116
##     1      68  52   120
##     Total 110 126   236
##
## $measure
##          odds ratio with 95% C.I.
## Predictor  estimate     lower     upper
##         0 1.0000000        NA        NA
##         1 0.4361502 0.2565763 0.7341383
##
## $p.value
##          two-sided
## Predictor  midp.exact fisher.exact  chi.square
##         0          NA           NA          NA
##         1 0.001724258  0.001793321 0.002532947
##
## $correction
## [1] TRUE
##
## attr(,"method")
## [1] "median-unbiased estimate & mid-p exact CI"
```

```
epitools::oddsratio(
  survey_data_clean$HTMFactor2,
  y = survey_data_clean$Univeristy_degree ,
  correction = T
)
```

```
## $data
##          Outcome
## Predictor   1   2 Total
##     0      53  58   111
##     1      57  68   125
##     Total 110 126   236
##
## $measure
##          odds ratio with 95% C.I.
## Predictor estimate     lower    upper
```

```
##         0 1.000000        NA       NA
##         1 1.089655 0.6514365 1.823854
##
## $p.value
##          two-sided
## Predictor midp.exact fisher.exact chi.square
##         0         NA           NA         NA
##         1  0.7433916    0.7942049   0.841948
##
## $correction
## [1] TRUE
##
## attr(,"method")
## [1] "median-unbiased estimate & mid-p exact CI"
```

```
epitools::oddsratio(
  survey_data_clean$HTMFactor3,
  y = survey_data_clean$Univeristy_degree ,
  correction = T
)
```

```
## $data
##          Outcome
## Predictor   1   2 Total
##     0      48  74   122
##     1      62  52   114
##     Total 110 126   236
##
## $measure
##          odds ratio with 95% C.I.
## Predictor  estimate     lower     upper
##         0 1.0000000        NA        NA
##         1 0.5459166 0.3235198 0.9150142
##
## $p.value
##          two-sided
## Predictor midp.exact fisher.exact chi.square
##         0         NA           NA         NA
##         1 0.02153792   0.02632906 0.02894874
##
## $correction
## [1] TRUE
##
## attr(,"method")
## [1] "median-unbiased estimate & mid-p exact CI"
```

```
epitools::oddsratio(
  survey_data_clean$HTMFactor4,
  y = survey_data_clean$Univeristy_degree ,
  correction = T
)
```

```
## $data
```

```
##          Outcome
## Predictor   1   2 Total
##     0      57  69   126
##     1      53  57   110
##     Total 110 126   236
##
## $measure
##          odds ratio with 95% C.I.
## Predictor  estimate     lower    upper
##         0 1.0000000        NA       NA
##         1 0.8889839 0.5308402 1.487327
##
## $p.value
##          two-sided
## Predictor midp.exact fisher.exact chi.square
##         0         NA           NA         NA
##         1  0.6538606    0.6955925  0.7478821
##
## $correction
## [1] TRUE
##
## attr(,"method")
## [1] "median-unbiased estimate & mid-p exact CI"
```

```
#Familiarity

epitools::oddsratio(
  survey_data_clean$HTMFactor1,
  y = survey_data_clean$X7_FAMILIARITY,
  rev = "columns",
  correction = T
)
```

```
## $data
##          Outcome
## Predictor   2  1 Total
##     0     121 48   169
##     1     124 44   168
##     Total 245 92   337
##
## $measure
##          odds ratio with 95% C.I.
## Predictor estimate     lower    upper
##         0 1.000000        NA       NA
##         1 0.895026 0.5521719 1.447982
##
## $p.value
##          two-sided
## Predictor midp.exact fisher.exact chi.square
##         0         NA           NA         NA
##         1  0.6511887    0.7140242  0.7387976
##
## $correction
## [1] TRUE
```

```
##
## attr(,"method")
## [1] "median-unbiased estimate & mid-p exact CI"
```

```
epitools::oddsratio(
  survey_data_clean$HTMFactor2,
  y = survey_data_clean$X7_FAMILIARITY,
  rev = "columns",
  correction = T
)
```

```
## $data
##          Outcome
## Predictor   2  1 Total
##     0     114 55   169
##     1     131 37   168
##     Total 245 92   337
##
## $measure
##          odds ratio with 95% C.I.
## Predictor  estimate     lower     upper
##         0 1.0000000        NA        NA
##         1 0.5870165 0.3584081 0.9530119
##
## $p.value
##          two-sided
## Predictor midp.exact fisher.exact chi.square
##         0         NA           NA         NA
##         1 0.03110712   0.03743202 0.04082482
##
## $correction
## [1] TRUE
##
## attr(,"method")
## [1] "median-unbiased estimate & mid-p exact CI"
```

```
epitools::oddsratio(
  survey_data_clean$HTMFactor3,
  y = survey_data_clean$X7_FAMILIARITY,
  rev = "columns",
  correction = T
)
```

```
## $data
##          Outcome
## Predictor   2  1 Total
##     0     122 47   169
##     1     123 45   168
##     Total 245 92   337
##
## $measure
##          odds ratio with 95% C.I.
## Predictor  estimate     lower   upper
```

```
##         0 1.0000000        NA      NA
##         1 0.9499147 0.5864886 1.53718
##
## $p.value
##          two-sided
## Predictor midp.exact fisher.exact chi.square
##         0         NA           NA         NA
##         1   0.834098    0.9027842  0.9291656
##
## $correction
## [1] TRUE
##
## attr(,"method")
## [1] "median-unbiased estimate & mid-p exact CI"
```

```
epitools::oddsratio(
  survey_data_clean$HTMFactor4,
  y = survey_data_clean$X7_FAMILIARITY,
  rev = "columns",
  correction = T
)
```

```
## $data
##          Outcome
## Predictor   2  1 Total
##     0     125 44   169
##     1     120 48   168
##     Total 245 92   337
##
## $measure
##          odds ratio with 95% C.I.
## Predictor estimate     lower   upper
##         0 1.000000        NA      NA
##         1 1.135661 0.7019993 1.84083
##
## $p.value
##          two-sided
## Predictor midp.exact fisher.exact chi.square
##         0         NA           NA         NA
##         1  0.6041442    0.6262623  0.6890035
##
## $correction
## [1] TRUE
##
## attr(,"method")
## [1] "median-unbiased estimate & mid-p exact CI"
```

```
#Deontology

epitools::oddsratio(
  survey_data_clean$HTMFactor1,
  y = survey_data_clean$Deont_Util_class,
  correction = T
)
```

```
## $data
##          Outcome
## Predictor Deontologically\nOriented Prone to Utilitarian\nThinking Total
##     0                           100                                69   169
##     1                            63                               105   168
##     Total                       163                               174   337
##
## $measure
##          odds ratio with 95% C.I.
## Predictor estimate   lower    upper
##         0 1.000000      NA       NA
##         1 2.406797 1.55678 3.746847
##
## $p.value
##          two-sided
## Predictor  midp.exact fisher.exact  chi.square
##         0          NA           NA          NA
##         1 7.12737e-05 8.392245e-05 0.000108179
##
## $correction
## [1] TRUE
##
## attr(,"method")
## [1] "median-unbiased estimate & mid-p exact CI"
```

```
epitools::oddsratio(
  survey_data_clean$HTMFactor2,
  y = survey_data_clean$Deont_Util_class,
  correction = T
)
```

```
## $data
##          Outcome
## Predictor Deontologically\nOriented Prone to Utilitarian\nThinking Total
##     0                            72                                97   169
##     1                            91                                77   168
##     Total                       163                               174   337
##
## $measure
##          odds ratio with 95% C.I.
## Predictor  estimate     lower     upper
##         0 1.0000000        NA        NA
##         1 0.6292368 0.4079592 0.9670611
##
## $p.value
##          two-sided
## Predictor midp.exact fisher.exact chi.square
##         0         NA           NA         NA
##         1 0.03458994   0.03849864 0.04392321
##
## $correction
## [1] TRUE
##
## attr(,"method")
```

```
## [1] "median-unbiased estimate & mid-p exact CI"
```

```
epitools::oddsratio(
  survey_data_clean$HTMFactor3,
  y = survey_data_clean$Deont_Util_class,
  correction = T
)
```

```
## $data
##          Outcome
## Predictor Deontologically\nOriented Prone to Utilitarian\nThinking Total
##     0                            95                                74   169
##     1                            68                               100   168
##     Total                       163                               174   337
##
## $measure
##          odds ratio with 95% C.I.
## Predictor estimate    lower    upper
##         0 1.000000       NA       NA
##         1 1.883081 1.223014 2.914096
##
## $p.value
##          two-sided
## Predictor  midp.exact fisher.exact  chi.square
##         0          NA           NA          NA
##         1 0.003993033  0.004580321 0.005412176
##
## $correction
## [1] TRUE
##
## attr(,"method")
## [1] "median-unbiased estimate & mid-p exact CI"
```

```
epitools::oddsratio(
  survey_data_clean$HTMFactor4,
  y = survey_data_clean$Deont_Util_class,
  correction = T
)
```

```
## $data
##          Outcome
## Predictor Deontologically\nOriented Prone to Utilitarian\nThinking Total
##     0                            82                                87   169
##     1                            81                                87   168
##     Total                       163                               174   337
##
## $measure
##          odds ratio with 95% C.I.
## Predictor estimate     lower    upper
##         0 1.000000        NA       NA
##         1 1.012319 0.6594304 1.554091
##
## $p.value
```

```
##          two-sided
## Predictor midp.exact fisher.exact chi.square
##         0         NA           NA         NA
##         1  0.9553964            1          1
##
## $correction
## [1] TRUE
##
## attr(,"method")
## [1] "median-unbiased estimate & mid-p exact CI"
```
